# Rapid Identification of the Tumor-Specific Reactive TIL Repertoire *via* Combined Detection of CD137, TNF, and IFNγ, Following Recognition of Autologous Tumor-Antigens

**DOI:** 10.3389/fimmu.2021.705422

**Published:** 2021-10-11

**Authors:** Arianna Draghi, Christopher Aled Chamberlain, Shawez Khan, Krisztian Papp, Martin Lauss, Samuele Soraggi, Haja Dominike Radic, Mario Presti, Katja Harbst, Aishwarya Gokuldass, Anders Kverneland, Morten Nielsen, Marie Christine Wulff Westergaard, Mads Hald Andersen, Istvan Csabai, Göran Jönsson, Zoltan Szallasi, Inge Marie Svane, Marco Donia

**Affiliations:** ^1^National Center for Cancer Immune Therapy (CCIT-DK), Department of Oncology, Copenhagen University Hospital, Herlev, Denmark; ^2^Department of Physics of Complex Systems, ELTE Eötvös Loránd University, Budapest, Hungary; ^3^Division of Oncology and Pathology, Department of Clinical Sciences Lund, Faculty of Medicine, Lund University, Lund, Sweden; ^4^Lund University Cancer Centre, Lund University, Lund, Sweden; ^5^Bioinformatics Research Center, Aarhus University, Aarhus, Denmark; ^6^Danish Cancer Society Research Center, Copenhagen, Denmark

**Keywords:** CD137 (4-1BB), immune-responses to cancer, tumor-specific activation, tumor-specific reactivity, single-cell technologies, tumor-infiltrating lymphocytes (TILs), immune-monitoring

## Abstract

Detecting the entire repertoire of tumor-specific reactive tumor-infiltrating lymphocytes (TILs) is essential for investigating their immunological functions in the tumor microenvironment. Current *in vitro* assays identifying tumor-specific functional activation measure the upregulation of surface molecules, *de novo* production of antitumor cytokines, or mobilization of cytotoxic granules following recognition of tumor-antigens, yet there is no widely adopted standard method. Here we established an enhanced, yet simple, method for identifying simultaneously CD8^+^ and CD4^+^ tumor-specific reactive TILs *in vitro*, using a combination of widely known and available flow cytometry assays. By combining the detection of intracellular CD137 and *de novo* production of TNF and IFNγ after recognition of naturally-presented tumor antigens, we demonstrate that a larger fraction of tumor-specific and reactive CD8^+^ TILs can be detected *in vitro* compared to commonly used assays. This assay revealed multiple polyfunctionality-based clusters of both CD4^+^ and CD8^+^ tumor-specific reactive TILs. *In situ*, the combined detection of *TNFRSF9*, *TNF*, and *IFNG* identified most of the tumor-specific reactive TIL repertoire. In conclusion, we describe a straightforward method for efficient identification of the tumor-specific reactive TIL repertoire *in vitro*, which can be rapidly adopted in most cancer immunology laboratories.

## 1 Introduction

Tumor-infiltrating lymphocytes (TILs) are a heterogeneous population of T cells comprising tumor-specific reactive T lymphocytes and bystander T cells (1). Recently, numerous promising attempts have been made to differentiate these populations comprehensively, either through markers of tumor specificity (i.e., CD39, CD103, PD1) (1–3) or tumor reactivity (*de novo* antitumor cytokine production, mobilization of cytotoxic granules, upregulation of activation markers following recognition of autologous tumor-antigens) (4–6). Although useful for various clinical applications, tumor specificity markers do not allow for real-time tumor reactivity detection (i.e., active recognition) as they only define potential, not active, recognition of tumor cells. In contrast, tumor reactivity markers facilitate the characterization of the ongoing immune response by detecting those cells engaged actively in tumor recognition.

The most common approaches for detecting and characterizing tumor-specific reactive TILs *in vitro* assess effector functions after specific stimulation with either pure tumor-antigens (e.g., peptides) or autologous tumor cells (naturally presenting tumor-antigens). TILs exerting one or more effector functions after recognition of tumor-antigens are commonly considered tumor-reactive, and the selection of tumor-reactive TILs for further processing in adoptive cell transfer protocols has the potential to improve patient responses (7, 8). In addition, the identification and subsequent functional and phenotypical characterization of the tumor-specific reactive TIL pool is an important avenue for improving and developing new immunotherapies. The most commonly used approach is intracellular cytokine staining (ICS) focusing on the detection of molecules such as interferon-gamma (IFNγ), tumor necrosis factor (TNF), and interleukin 2 (IL-2) (6, 9, 10), after recognition of tumor-antigens. Importantly, ICS is often combined with the detection of additional effector molecules or markers upregulated after T cell activation, such as granzyme B (GZMB) (11), the widely used marker of T cell degranulation CD107a (lysosomal-associated membrane protein-1/LAMP-1) (5), or the CD4^+^ T cell-specific surface activation marker CD154 (CD40L) (12). Other recent studies have indicated that activation-induced upregulation of CD137 (also known as 4-1BB, encoded by *TNFRSF9*) could be a more comprehensive marker for identifying antigen-specific reactive T cells (4, 13–18).

Overall, no standard method has been universally adopted and it is still unclear whether merging current methods is feasible and can improve the detection of the entire tumor-specific reactive TIL repertoire. To maximize detection whilst maintaining an easy-to-use assay, we leveraged multiple data sources to develop and validate a rapid and straightforward protocol. By using a combination of three functional biomarkers commonly upregulated following T cell receptor (TCR)-mediated recognition of naturally presented autologous tumor-antigens, this protocol identifies a larger fraction of the tumor-specific reactive TIL population *in vitro* compared to standard methods.

## 2 Materials and Methods

### 2.1 Immune Cells and Tumors

#### 2.1.1 Sample Origin

Tumor samples were obtained *via* biopsy collection for enrollment in clinical trials at the National Center for Cancer Immune Therapy (CCIT-DK), Department of Oncology, Copenhagen University Hospital, Herlev, Denmark. Sample processing, including the generation of cell lines and TIL cultures, was performed in CCIT-DK trials whose primary results were already reported (19–23), or in trial H-19076238. Only samples where TILs had a high percentage of tumor-reactive TILs in previous studies (24) were selected. Twenty-three metastatic melanoma (MM), one ovarian cancer (OC) and one sarcoma (SAR) samples were used for this study. All samples were derived from treatment naïve patients, except for 11 MM samples derived from patients previously treated with checkpoint-inhibitor immunotherapy. All procedures were performed in compliance with the clinical protocols approved by the Ethics Committee of the Capital Region of Denmark and national regulations for biomedical research.

#### 2.1.2 Immune Cells

TILs were isolated and expanded *in vitro* from tumor fragments with a two-step process in previous studies (19–23). Young TILs were obtained after minimal-culture, whereas rapid-expansion protocol (REP) TILs were obtained after massive expansion (25). All TILs not otherwise enriched or purified were named “bulk” TILs.

#### 2.1.3 Tumor Cell Lines (TCLs)

Autologous short-term *in vitro* cultured TCLs were established *via* serial passage of adherent cells from tumor fragments derived from the same tumor lesion from which the TILs were generated, as described previously (25). All TCLs were generated internally and authenticated *via in vitro* patterns of growth, morphology (light microscopy), and when in doubt, expression of lineage antigens by PCR. Mycoplasma testing (Cat. No A3744.0020, VWR International, Lutterworth, UK) was performed following the manufacturer’s instructions and was negative. To abrogate the expression of major histocompatibility complex (MHC) class I or class II, as previously described (26, 27), selected TCLs were subjected to clustered regularly interspaced short palindromic repeats (CRISPR)/CRISPR-associated protein 9 (Cas9)-mediated knockout of *B2M* or *CIITA*. crRNAs targeting the *B2M* (5´-CAGTAAGTCAACTTCAATGT-3’) and *CIITA* (5´-GATATTGGCATAAGCCTCCC-3`) genes were selected from the literature (26) or designed using the Custom Alt-R^®^ CRISPR-Cas9 guide RNA design tool (Integrated DNA Technologies, Coralville, IA, US). crRNAs, tracrRNA, and S.p. HiFi Cas9 Nuclease V3 were purchased from Integrated DNA Technologies’ Alt-R catalog, and ribonucleoprotein (RNP) complexes were formed as per the manufacturer’s instructions. 125,000 tumor cells were then seeded in a well of a 48-well plate and transfected with RNP complexes at a concentration of 30nM using Lipofectamine™ CRISPRMAX™ Cas9 Transfection Reagent (Cat No CMAX00008, Thermo Fisher Scientific, Waltham, MA, USA) according to the manufacturer’s instructions. After at least 72hrs, loss of MHC class I or II was verified by flow cytometry, and purified *B2M* KO or *CIITA* KO populations were generated by electronic sorting on a FACS Aria (BD Biosciences, Kongens Lyngby, Denmark) and further expansion. Details of the flow cytometry antibodies can be found in the **Supplementary Methods**.

### 2.2 Enrichment or Purification of TILs

#### 2.2.1 Enrichment of CD8^+^ and CD4^+^ Bulk TILs

Where indicated, CD8^+^ or CD4^+^ Young TILs were enriched using CD8 or CD4 MicroBeads (Cat No 130-045-201 and 130-045-101, Miltenyi Biotec, Bergisch Gladbach, Germany) according to manufacturer instructions. “Enriched CD8^+”^ or “enriched CD4^+^” Young TILs were subsequently expanded with the rapid expansion protocol (25).

#### 2.2.2 Generation of Pure CD8^+^ TILs Specific for Known Autologous Tumor-Antigens

CD8^+^ TIL cultures of known antigen specificity were obtained in previous internal studies using fluorochrome-conjugated peptide-MHC class I-tetramer-based sorting as described previously (28). These CD8^+^ TILs were >95% specific for a known MHC class I-restricted epitope (minimal peptide) derived from a tumor-antigen recognized on the autologous tumor cells. CD8^+^ TILs specific for Melanoma-associated antigen recognized by T cells 1 (MART-1), Interferon-inducible protein (AIM-2), Melanoma-associated antigen 1 (MAGE-A1), or a neo-antigen derived from the gene *USP34* were used.

#### 2.2.3 Enrichment of Tumor-Reactive CD8^+^TILs

CD8^+^ TIL cultures comprising a polyclonal population of highly tumor-specific TILs, but targeting unknown antigens presented by autologous tumor cells, were obtained by electronic sorting on a FACS Aria (BD) of CD8^+^ Young TILs upregulating CD137 following recognition of autologous tumor cells. The sorted CD8^+^ TILs were further expanded as described previously (28).

### 2.3 Preparation for T Cell Activation Assays

TILs were thawed and rested overnight at 37°C in TIL media (RPMI-1640 plus GlutaMAX and 25mM HEPES (Cat. No 72400-021, Gibco, Thermo Fisher Scientific) supplemented with 10% heat-inactivated human AB serum (H4522, Sigma-Aldrich/Merck KGaA, Darmstadt, Germany), 100 U/ml penicillin, and 100 µg/ml streptomycin (Pen Strep, Cat No 15140122, Gibco, Thermo Fisher Scientific)) before being washed and stimulated. Prior to co-culture assays, TCLs were pre-treated for 72 hours with 100 IU/ml IFNγ to increase antigen presentation and cultured at 37°C in RPMI 1640 plus GlutaMAX and 25mM HEPES supplemented with 10% FCS, 100 U/ml penicillin, and 100 µg/ml streptomycin.

### 2.4 T Cell Activation Assays

All experiments were performed using autologous matched pairs of TILs and TCLs to reproduce the natural presentation of tumor-antigens occurring in the tumor microenvironment (TME) of individual patients. Tumor-specific immune activation was assessed with 8-hour TIL-autologous TCL (effector/target ratio of 3:1) co-culture assays at 37°C. In selected flow cytometry experiments, anti-CD107a antibody was added at the beginning of the co-culture assay. Brefeldin A (BFA) (1:1000 dilution, GolgiPlug™, Cat No 555029, BD) and/or Monensin (MN) (1:1000 dilution, GolgiStop™, Cat No 554724, BD) were used when indicated. TILs alone or co-incubated with allogeneic TCLs (shown in **Supplementary Figures 1A, B**) served as negative controls. A pre-screening was performed using TILs and multiple allogeneic TCLs from our institution’s cell line bank to select TCLs without alloreactivity and thereby rule out any unspecific T cell activation by irrelevant TCLs. Only TILs where no upregulation of functional markers was detected when co-culturing with at least one allogeneic TCL were used in this study. The dependence of functional marker upregulation on TCR-MHC-mediated recognition of autologous tumor-antigens was confirmed by disrupting MHC class I or MHC class II on the surface of autologous TCLs in selected samples, resulting in abrogation of CD8^+^ and CD4^+^ T cell recognition, respectively (TNF and IFNγ are shown in **Supplementary Figures 1C, D**; CD137 and CD107a not shown). The 8-hour time point was chosen based on our previous data regarding CD137 (29) and the reported optimal incubation time to detect TNF and IFNγ [6 to 12 hours (10, 30)]. The expression kinetics of the activation markers were verified in preliminary experiments (**Supplementary Figure 2**).

### 2.5 Bulk Transcriptomic Analysis

Twelve CD8^+^ (seven enriched CD8^+^ bulk TIL cultures (MM), two pure CD8^+^ TIL cultures specific for known autologous tumor-antigens (MM), and three enriched tumor-reactive TIL cultures (1 MM, 1 OC, 1 SAR)) and eleven enriched CD4^+^ bulk TIL cultures (MM), obtained from distinct individual patients and selected for high reactivity to tumor-antigens, were isolated post-co-culture with autologous or allogeneic TCLs using CD8 or CD4 MicroBeads according to the manufacturer’s instructions, and stored in RNAlater (Cat No R0901, Sigma-Aldrich). RNA was extracted from collected TILs using an AllPrep DNA/RNA Mini Kit (Cat No 80204, Qiagen, Hilden, Germany) and RNA sequencing was performed on a NextSeq500 (Illumina, San Diego, California, USA) as previously described (31). Log_2_-fold changes (LFC) in gene expression between TILs co-cultured with autologous tumor cells and TILs co-cultured with allogeneic tumor cells were calculated by subtracting the gene-expression values between these experimental conditions after standard normalization and log-transformation. Tumor-specific activation gene sets were generated by first identifying the differences in gene expression (other than *TNFRSF9*, *TNF*, and *IFNG*) between TILs exposed to autologous tumor-antigens and TILs exposed to allogeneic-tumor antigens (control). These differentially expressed genes (DEGs) were then filtered according to p-value (<0.01) and mean LFC (>2, CD8^+^ TILs; >1.4, CD4^+^ TILs).

### 2.6 Flow Cytometry Staining Assays

After incubation cells were collected, washed twice with Dulbecco’s phosphate-buffered saline (DPBS, Cat No D8537, Sigma-Aldrich/Merck KgaA), and stained with live/dead reagent and antibodies for surface markers (**Supplementary Table 1**). The cells were then washed, fixed, and permeabilized overnight at 4°C using the FoxP3/Transcription Factor Staining Buffer Set (Cat No 00-5523, eBiosciences, Thermo Fisher Scientific). The following day cells were stained with antibodies binding intracellular targets (**Supplementary Table 1**). After staining and washing, cells were resuspended in 100 ul of DPBS and analyzed on a BD FACSCanto™ II Flow Cytometer (BD), BD™ LSR II Flow Cytometer (BD), or NovoCyte Quanteon™ Flow Cytometer (Agilent, Santa Clara, CA, USA). Details on the flow cytometry antibodies used in the study can be found in the **Supplementary Methods**.

### 2.7 Flow Cytometry Data Processing

In tests evaluating the differential expression of CD137 on activated TILs in the presence or absence of BFA and/or MN, TIL activation was defined as the percentage of live T cells staining positive for either surface expression of CD137 (S-CD137), intracellular expression of CD137 (IC-CD137), or at least one form of CD137 (Total CD137 or T-CD137), minus control (TILs alone). Boolean gating “S-CD137 OR IC-CD137” was performed to obtain the “T-CD137” population. Bulk MM TIL samples were used for these analyses.

In tests evaluating CD137 staining in combination with the detection of TNF, IFNγ, and CD107a, TIL activation was defined in three distinct ways: 1) as the percentage of live TILs staining positive for CD137 (“CD137^+^ TILs”), 2) as the percentage of live TILs staining positive for at least one of TNF, IFNγ, and CD107a (“Antitumor Function^+^ TILs”), or 3) as the percentage of live TILs staining positive for at least one of CD137, TNF, IFNγ, and CD107a (“Total Reactive TILs”), minus control (TILs alone). Bulk TILs or enriched CD8^+^ and CD4^+^ bulk TILs were used for the data presented in section *3.3 Combined Detection of CD137, TNF, and IFNγ Enhances the Detection of Tumor-Specific Reactive Bulk TILs on a Protein Level In Vitro* (19 MM, 1 OC and 1 SAR samples for CD8^+^ TILs and 16 MM samples for CD4^+^ TILs). Two pure CD8^+^ TIL samples (MM) specific for a tumor-neoantigen and for MAGE-A1, respectively, were used for the data presented in *3.4 Pure Tumor Antigen-Specific CD8^+^ TILs Exhibit Tumor-Reactivity Profiles Comparable to Bulk TILs on a Protein Level In Vitro*. Flow cytometry data analysis, including the generation of t-SNE plots, was performed using FlowJo V10 (see **Supplementary Figure 3** for gating strategies). For multifunctional characterization analyses of tumor-specific reactive TILs, FlowJo V10 boolean gating generated 7 (excluding the CD137^-^/TNF^-^/IFNγ^-^ population) unique combinations of the three analyzed markers (CD137, TNF, IFNγ), and quantified the percentage of CD8^+^ or CD4^+^ TILs belonging to each group. Data were exported, and the background subtracted.

### 2.8 T Cell Transcriptomics Single-Cell Data From Public Repositories

We collected T cell single-cell transcriptomics data from numerous studies deposited in public repositories by surveying the literature to identify relevant single-cell RNA sequencing (scRNAseq) datasets. Only those containing T cell data from human tumor biopsies and using the Smart-seq2 protocol for library preparation were considered. Based on this criterion, we identified six datasets from five independent studies (GSE98638, GSE99254, GSE108989, GSE115978, GSE120575) containing scRNAseq data from 112 tumor biopsies collected from 94 patients, across a total of four tumor types [14 non-small cell lung (32)-, 6 hepatocellular (33)-, 12 colorectal (34)-cancer, and 62 melanoma (35–37)]. All heatmaps and tSNE plots from scRNAseq data were produced using the open-source software BIOMEX (version 1.0-1) (38). Detailed information can be found in **Supplementary Methods**, **Supplementary Table 2**, and **Supplementary Table 3**.

### 2.9 Statistical Analysis

Statistical tests were conducted using a paired Wilcoxon signed-rank test unless otherwise described. Graphs and statistical analyses were generated using GraphPad Prism 9.0.0. Negative values deriving from the subtraction of unstimulated samples from stimulated samples were converted to 0.01% for statistical analyses and generation of figures. Values exceeding 100% after normalization due to previous background subtraction were converted to 100% for statistical analyses and generation of figures. All values were expressed as median unless otherwise specified. Statistical tests related to DEGs were conducted using a paired t-test.

## 3 Results

### 3.1 TILs Upregulate Genes Coding for Common Tumor Reactivity Markers Following Recognition of Autologous Tumor-Antigens

We initially searched for markers associated with tumor-antigen recognition that could be used in both main subpopulations of TILs (CD8^+^ and CD4^+^ TILs). Hence, we investigated whether genes coding for some of the most commonly used T cell activation molecules (TNF, IFNγ, IL-2, CD107a, CD154, CD137, GZMB) were upregulated in in-house bulk RNA sequencing data obtained from 12 CD8^+^ (10 MM, 1 SAR, 1 OC) and 11 CD4^+^ enriched TIL samples (MM) following recognition of naturally-presented autologous tumor antigens. Of the common T cell activation molecules considered, only *TNF* (TNF)*, IFNG* (IFNγ), *TNFRSF9* (CD137), and *GZMB* (GZMB) were significantly upregulated (LFC > 1, p-value<0.01) in both CD8^+^ and CD4^+^ TILs following recognition of autologous TCLs, whereas the mean LFCs of *IL2* (IL-2), *LAMP1* (CD107a), and *CD40LG* (CD154) approached zero (**Supplementary Table 4**). Although significantly upregulated at the mRNA level post-stimulation, GZMB protein is in fact continuously synthesized and stored in an inactive form by resting T cells (39, 40). This resulted in excessive background noise limiting its use as a discriminatory marker in flow cytometry and in scRNAseq data (internal data, not shown). Hence, only *TNF* (TNF)*, IFNG* (IFNγ), and *TNFRSF9* (CD137) were shortlisted for further testing.

### 3.2 Intracellular Transport Inhibition Is Compatible With Intracellular but Not Surface Detection of CD137 *In Vitro*

**Figure 1:**
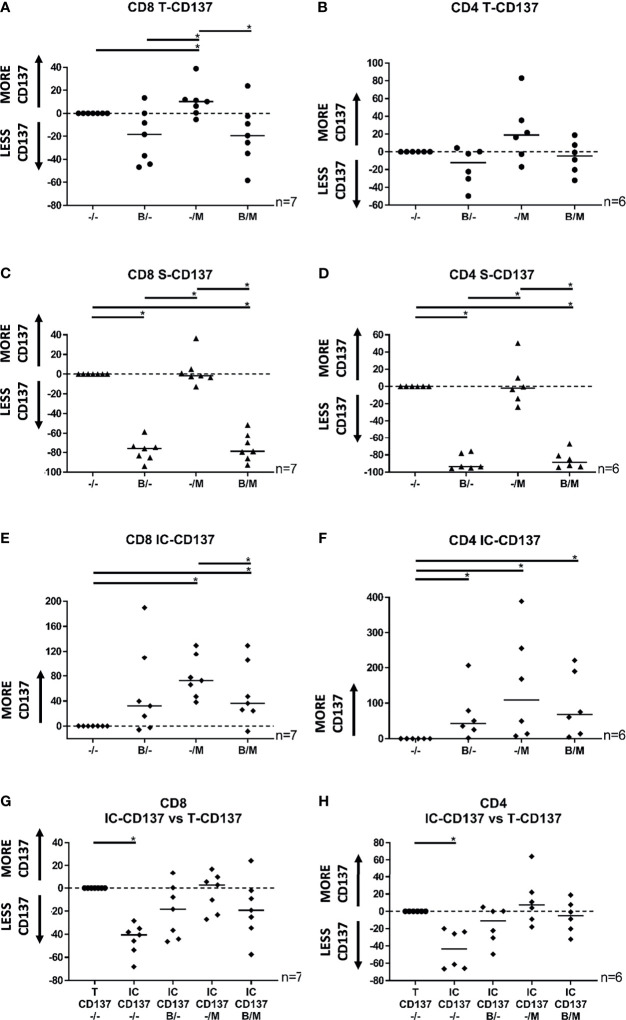
Scatter plots showing the total, surface, and intracellular expression of CD137 in CD8^+^ and CD4^+^ TILs following 8 hours of co-culture with autologous tumor cells. **(A)** Total CD137 expression in CD8^+^ TILs in the presence of BFA, MN, or the combination of BFA and MN was modulated by -18%, +10%, and -19%, respectively. **(B)** Total CD137 expression in CD4^+^ TILs in the presence of BFA, MN, or the combination of BFA and MN was modulated by -12%, +19%, and -5%, respectively. **(C)** Surface CD137 expression in CD8^+^ TILs in the presence of BFA, MN, or the combination of BFA and MN was modulated by -76%%, -2%, and -79%, respectively. **(D)** Surface CD137 expression in CD4^+^ TILs in the presence of BFA, MN, or the combination of BFA and MN was modulated by -94%, -2%, and -88%, respectively. **(E)** Intracellular CD137 expression in CD8^+^ TILs in the presence of BFA, MN, or the combination of BFA and MN was modulated by +32%, +73%, and +36%, respectively. **(F)** Intracellular CD137 expression in CD4^+^ TILs in the presence of BFA, MN, or the combination of BFA and MN was modulated by median +43%, +109%, and +68%, respectively. **(G, H)** Intracellular CD137 expression in **(G)** CD8^+^ TILs and **(H)** CD4^+^ TILs in the presence of BFA, MN, or the combination of BFA and MN was not significantly reduced in comparison to the total CD137 expression. TIL activation was defined as the percentage of live T cells staining positive for either surface expression of CD137 (S-CD137), intracellular expression of CD137 (IC-CD137), or at least one form of CD137 (Total CD137 or T-CD137), minus control (TILs alone). Normalized values relative to the relevant (surface, intracellular or total) CD137 expression in the absence of BFA and MN (-/-) are shown in the dot plots. Bulk MM TIL samples were used for these analyses. *p < 0.05; M, monensin, MN; B, brefeldin A, BFA.

Intracellular detection of common antitumor cytokines such as TNF and IFNγ requires the use of protein transport inhibitors. Therefore, to establish a protocol for simultaneous *in vitro* detection of CD137 upregulation and production of TNF and IFNγ, we initially tested how the protein transport inhibitors Brefeldin A (BFA) and Monensin (MN) affected the expression of CD137 in TILs following tumor-antigen specific activation (**Figure 1** and **Supplementary Figure 4**). BFA alone and the combination of BFA and MN did not affect the total (**Figures 1A, B**) but reduced the surface (**Figures 1C, D**) expression of CD137 in both CD8^+^ and CD4^+^ TIL subsets. In contrast, total and surface expression of CD137 was not reduced by MN alone (**Figures 1A–D**). All protein transport inhibitor combinations either increased or did not affect the intracellular expression of CD137 (**Figures 1E, F**). Overall, a reliable representation of total CD137 expression in a set-up compatible with TNF and IFNγ detection was achieved *via* intracellular staining of CD137 with all protein transport inhibitor combinations (**Figures 1G, H**). Importantly, intracellular detection of CD137 has previously been used to successfully detect activated CD8^+^ T cells (15).

### 3.3 Combined Detection of CD137, TNF, and IFNγ Enhances the Detection of Tumor-Specific Reactive Bulk TILs on a Protein Level *In Vitro*

Previous studies have demonstrated that biomarkers linked to T cell activation, but not *de novo* mRNA expression, such as CD107a (5, 41) and CD154 (42–44), can be used to detect tumor-antigen specific TIL-activation. We reasoned that these biomarkers could improve our panel if detected simultaneously with CD137, TNF, and IFNγ. However, the upregulation of CD154 can only be identified with MN alone (12), a condition that severely hampers the detection of TNF (10, 30). Hence, we tested whether the combined intracellular detection of CD137, TNF, and IFNγ was feasible, and whether the addition of CD107a to the panel enhanced the detection of tumor-specific reactive TILs *in vitro*. Based on our data, and on previous studies (10, 30) showing BFA to be optimal for detecting TNF and IFNγ, and MN to be optimal for detecting CD107a, the combination of BFA and MN was chosen to maximize the detection of tumor-specific reactive TILs on a protein level by intracellular staining after exposure to autologous TCLs.

**Figure 2:**
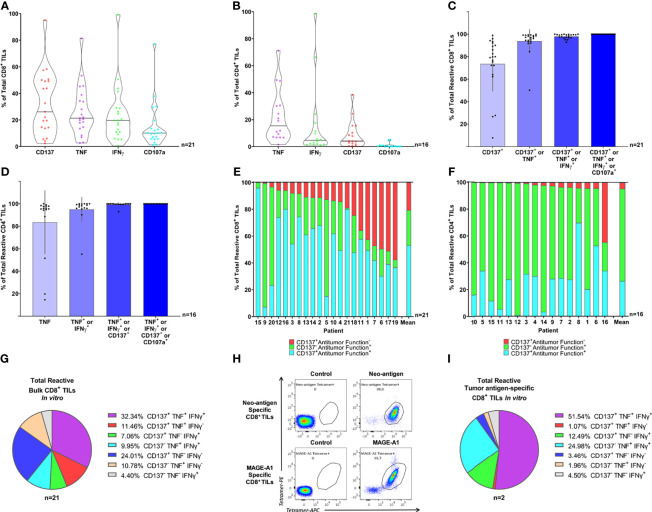
Combined intracellular detection of CD137, TNF, and IFNγ enhanced the detection of tumor-specific reactive bulk TILs *in vitro*. **(A, B)** Violin plots illustrate the expression of CD137, TNF, IFNγ, and CD107a in **(A)** CD8^+^ and **(B)** CD4^+^ TILs following 8 hours of co-culture with autologous TCLs, minus control (TILs alone). Individual markers are sorted by expression. Horizontal lines illustrate median values. **(C, D)** The relative individual contribution of the four markers analyzed (CD137, TNF, IFNγ, and CD107a, summed in order of relevance) to the total **(C)** CD8^+^ and **(D)** CD4^+^ TIL tumor-specific reactive population. Columns illustrate mean values and black bars represent standard deviations. Only cells expressing at least one of the four markers analyzed (CD137, TNF, IFNγ, and CD107a) are displayed. **(E, F)** Relative distribution of each distinct sub-population identified *via* combined detection of CD137, TNF, IFNγ, and CD107a within the **(E)** CD8^+^ and **(F)** CD4^+^ tumor-specific reactive TIL repertoire after 8 hours of co-culture with autologous TCLs. TNF, IFNγ, and CD107a were defined “Antitumor functions”, therefore, TILs were defined Antitumor Function^+^ if staining positive for at least one of TNF, IFNγ, and CD107a. Each bar represents an individual patient or the mean value. Only cells expressing at least one of the four markers analyzed (CD137, TNF, IFNγ, and CD107a) are displayed. **(G)** Relative distribution of the seven combinations of the three markers of interest (CD137, TNF, and IFNγ) within “bulk” tumor-specific reactive CD8^+^ TILs. Every pie chart slice represents a different combination of CD137, TNF, and IFNγ. TILs were gated on cells expressing at least one of the three markers analyzed (CD137, TNF, and IFNγ). The pie chart illustrates mean values. **(H)** Neo-antigen-specific CD8^+^ TILs (upper panel) and MAGE-A1-specific CD8^+^ TILs (lower panels) from two distinct patients were sorted based on 2-color tetramer binding and expanded *in vitro* followed by tetramer staining showing high specificity (>95%) for the autologous tumor-antigens. **(I)** The pie chart illustrates the relative distribution of the seven combinations of the three markers of interest (CD137, TNF, and IFNγ) within tumor-specific reactive CD8^+^ TILs, from pure tumor antigen-specific CD8^+^ TILs. Each pie chart slice represents a different combination of CD137, TNF, and IFNγ. Bulk TILs or enriched CD8^+^ and CD4^+^ bulk TILs were used to obtain the intracellular staining data presented in panel A-G (19 MM, 1 OC and 1 SAR samples for CD8^+^ TILs and 16 MM samples for CD4^+^ TILs) and only samples with high percentage of tumor-reactive TILs were selected for these analyses. Two pure CD8^+^ TIL samples specific for known autologous tumor-antigens (MM) were used to obtain the flow cytometry data presented in panel H (tetramer staining) and I (intracellular staining). The CD137^+^TNF^-^IFNγ^-^ population represented 24% of the bulk tumor-specific reactive CD8^+^ TILs. This suggested that the use of CD137 improved the detection of “bulk” tumor-specific reactive CD8^+^ TILs compared to the combination of TNF and IFNγ. The use of CD107a did not add value to the overall significance of the assay. The optimized detection of tumor-specific reactive TILs identifies multiple functional clusters of tumor-specific reactive TILs both in “bulk” and pure tumor antigen-specific CD8^+^ TILs *in vitro*.

We initially analyzed the performance of each marker separately (**Figures 2A, B**). CD137 detected the greatest number of reactive CD8^+^ TILs, followed by TNF, IFNγ, and CD107a, with CD137 alone identifying 74% of the total CD8^+^ reactive TILs on average (**Figures 2A, C**). In contrast, TNF was the most effective biomarker in the CD4^+^ subset, identifying up to 84% of total CD4^+^ reactive TILs on average (**Figures 2B, D**). Interestingly, the contribution of CD107a to identifying the repertoire of tumor-specific reactive TILs was negligible in both the CD8^+^ and CD4^+^ TIL compartments (**Figures 2C, D**). Overall, as only TILs with high level of recognition were used, the mean percentage of “bulk” TILs that were reactive to autologous tumor cells was high (measured with upregulation of CD137, TNF, IFNγ or CD107a: 41% in CD8^+^ TILs, n=21; and 29% in CD4^+^ TILs, n=16. Data not shown).

When sub-grouping the total tumor-specific reactive TILs based on upregulation of CD137 only (CD137^+^ Antitumor function^-^), upregulation/mobilization of at least one among TNF, IFNγ, and CD107a without CD137 (CD137^-^ Antitumor function^+^), or both (CD137^+^ Antitumor function^+^), we observed that all three populations were well represented within the total reactive CD8^+^ TILs: on average, 21% were CD137^+^ Antitumor function^-^, 26% were CD137^-^ Antitumor function^+^, and 53% were CD137^+^ Antitumor function^+^ (**Figure 2E** and **Supplementary Figure 5A**). In contrast, tumor-reactive CD4^+^ TILs were primarily represented by the CD137^-^ Antitumor function^+^ (average 69%) and CD137^+^ Antitumor function^+^ (average 26%) populations (**Figure 2F**), indicating that CD137 is critical for identifying the CD8^+^ but not the CD4^+^ reactive TIL repertoire. Interestingly, a large fraction of reactive CD8^+^ and CD4^+^ TILs (average 35% and 56% respectively) were positive for only a single marker (**Supplementary Figures 5B, C**). The relative distribution of all CD137, TNF, or IFNγ combinations is shown in **Figure 2G** (tumor-specific reactive CD8^+^ TILs) and **Supplementary Figure 5D** (tumor-specific reactive CD4^+^ TILs). Of note, the CD137^+^TNF^-^IFNγ^-^ population represented 24% of the bulk tumor-specific reactive CD8^+^ TILs (**Figure 2G**).

Overall, the combination of CD137, TNF, and IFNγ *via* intracellular detection was feasible and the use of CD137 improved the detection of tumor-specific reactive CD8^+^ TILs by more than 20% compared to the combination of TNF, IFNγ; and CD107a. The use of CD107a did not add value to the overall significance of the assay, whereas CD137 facilitated a more detailed characterization of TIL functions; a fraction of CD8^+^ TILs were reactive without performing common antitumor functions. Importantly, despite the CD137 staining only minimally improving the detection of tumor-specific reactive CD4^+^ TILs, the assay effectively detected both CD8^+^ and CD4^+^ tumor-reactive TILs simultaneously.

### 3.4 Pure Tumor Antigen-Specific CD8^+^ TILs Exhibit Tumor-Reactivity Profiles Comparable to Bulk TILs on a Protein Level *In Vitro*

Bystander T cells activated independently of tumor antigens are known to infiltrate solid tumors (1, 2, 45). Therefore, we investigated whether the functional profiles observed in highly polyclonal “bulk” TILs recognizing autologous TCLs could be reproduced in a setting with known TCR-MHC class I-mediated tumor recognition. To do so, we assessed by intracellular staining the responses of two distinct CD8^+^ TIL samples, purified for known tumor-antigen specificity (**Figure 2H**), after exposure to autologous TCLs. As expected, the number of “Total reactive TILs” within the two highly purified TIL cultures was particularly high, 85% and 90% respectively (data not shown), highlighting the sensitivity of the assay. Similar activation-associated functional phenotypes previously seen in “bulk” TILs (unique combinations of CD137/TNF/IFNγ) were observed (**Figure 2I**). Hence, we confirmed that the *bona fide* tumor-reactive clusters we observed in our “bulk” TIL populations were not caused by bystander activation. Moreover, only a fraction of CD8^+^ tumor-specific reactive TILs were simultaneously positive for all three markers, confirming that tumor-specific reactivity does not guarantee a uniform functionality profile at the single-cell level.

### 3.5 Tumor-Specific Reactive T Cell Transcriptomics Generates Representative CD8 and CD4 Activation Gene Sets

To generate an activation gene set that could be used as a proxy of tumor-specific TIL activation, we analyzed the entire transcriptome of TILs following recognition of autologous TCLs [in-house bulk RNA sequencing data obtained from 12 CD8^+^ (10 MM, 1 SAR, 1 OC) and 11 CD4^+^ enriched TIL samples (MM)]. Overall, we identified 40 and 27 DEGs (other than *TNFSRF9*, *TNF* and *IFNG*) for CD8^+^ and CD4^+^ TILs respectively, with 18 DEGs shared between the CD8 and CD4 activation gene sets (**Supplementary Table 5**). To confirm that these tumor-specific activation gene sets were associated with TCR-MHC-mediated tumor-recognition, and not bystander TIL activation, we verified that the same genes were highly upregulated in two pure tumor-antigen-specific CD8^+^ TILs co-cultured with autologous TCLs (>95% specificity for either AIM-2 or MART-1 antigens). Of the CD8 tumor-specific activation gene set, 93% and 80% of genes could be found within the 300 genes most upregulated by the two antigen-specific TIL populations, respectively, with a mean LFC >1 for 93% and 75% of the genes (**Supplementary Figure 6**). In conclusion, these activation gene sets were strongly associated with TIL recognition of naturally-presented autologous tumor-antigens.

### 3.6 *TNFRSF9, TNF*, and *IFNG* Identify the Majority of the Presumed Tumor-Specific Reactive TIL Repertoire *In Situ*

**Figure 3:**
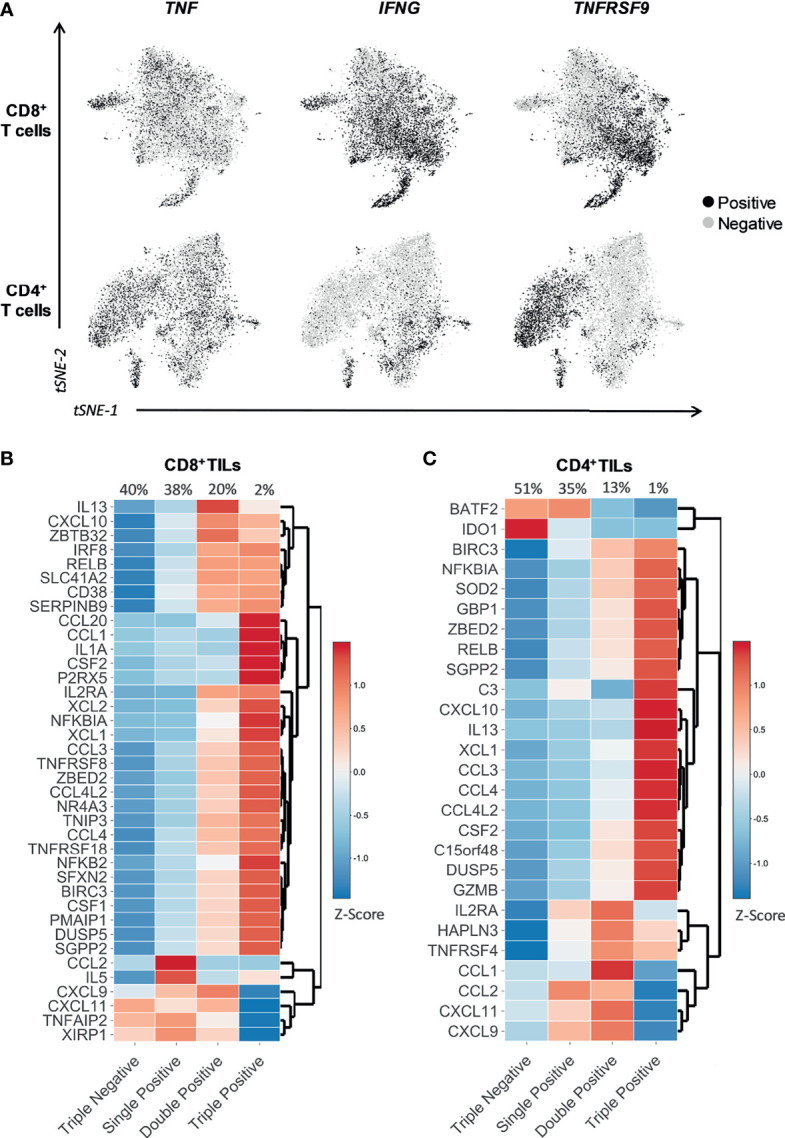
*TNFRSF9, TNF*, and *IFNG* allow the identification of tumor-specific reactive TILs *in situ*. t-SNE plots and heatmaps showing data from 12748 single CD8^+^ TILs and 10654 single CD4^+^ TILs isolated from tumor tissue of four different cancer types (non-small-cell lung cancer, hepatocellular carcinoma, colorectal cancer, and melanoma). Six selected scRNAseq datasets from public repositories were re-analyzed to produce this figure (see **Supplementary Table 2**). **(A)** Expression of the gene indicated above each plot, represented as black for positive expression and grey for negative. *TNFRSF9, TNF*, and *IFNG* were differentially expressed within both CD8^+^ and CD4^+^ TIL subsets. **(B, C)** The heatmaps show the expression (Z-score) of the genes belonging to the **(B)** CD8 and **(C)** CD4 tumor-specific activation gene sets within four functional clusters of CD8^+^ and CD4^+^ TILs identified *in situ* through the differential expression of *TNFRSF9, TNF*, and *IFNG*. The four functional clusters are characterized by the expression of either zero (Triple Negative), one (Single Positive), two (Double Positive), or three (Triple Positive) of the three genes of interest (*TNFRSF9, TNF*, and *IFNG*). *TNFRSF9, TNF*, and *IFNG* represent an efficient and effective surrogate combination for a much broader panel of functions associated to tumor reactivity, and can potentially identify the majority of the tumor-specific reactive TIL repertoire *in situ*.

To verify whether the expression of *TNFRSF9*, *TNF*, and *IFNG* could identify all TILs actively engaged in tumor recognition *in situ*, we re-analyzed multiple scRNAseq datasets using fresh tumor biopsies (**Supplementary Figure 7**) (32–37) and applied the activation gene sets as a proxy of presumed ongoing antigen stimulation in the TME. The level of expression of *TNFRSF9, TNF*, and *IFNG* was largely heterogeneous across both the CD8^+^ and the CD4^+^ TIL compartments (**Figure 3A**). In both the CD8^+^ and CD4^+^ TIL subsets, we observed that the expression of genes in the activation gene sets increased as reactivity marker (CD137, TNF, or IFNγ) gene positivity increased, i.e., the highest expression values for the majority of tumor-specific activation genes were observed in the Triple Positive cluster (*TNFRSF9*^+^ *TNF*^+^ *IFNG*^+^). In contrast, the Triple Negative cluster (*TNFRSF9*^-^ *TNF*^-^ *IFNG*^-^) demonstrated comparatively lower expression values for nearly all selected genes (**Figures 3B, C**). **Supplementary Figures 8A, B** illustrate the expression of the activation gene sets across all the functional clusters identified by distinct combinations of *TNFRSF9, TNF*, and *IFNG*. A positive gradient of exhaustion/dysfunction was observed from the Triple Negative cluster to the Triple Positive cluster (**Supplementary Figures 8C, D**), suggesting that cells expressing multiple functional markers of tumor reactivity could be more exhausted/dysfunctional than those expressing fewer functional markers. *TNFRSF9* had the highest level of association with exhaustion/dysfunction, with the greatest level of exhaustion/dysfunction exhibited by TILs expressing *TNFRSF9, TNF*, and/or *IFNG* (**Supplementary Figures 8C, D**). Expression of T cell co-stimulatory and effector molecule genes increased from the Triple Negative cluster to the Triple Positive cluster (**Supplementary Figures 9A, B**). However, TILs expressing *TNFRSF9* had the highest level of association with genes coding for co-stimulatory molecules, whilst those expressing *IFNG* associated with the expression of genes coding for effector-molecules (**Supplementary Figures 9A, B**). In contrast, naïve T cell markers were most associated with the Triple Negative and only *TNF^+^* cluster (**Supplementary Figures 9A, B**).

In summary, *TNFRSF9, TNF*, and *IFNG* represent an efficient and effective surrogate combination for a much broader panel of functions associated to tumor-reactivity, and can potentially identify the majority of the tumor-specific reactive TIL repertoire *in situ*. Additionally, multiple distinct functional clusters of tumor-specific reactive CD8^+^ and CD4^+^ TILs observed *in vitro* were also present *in situ*.

### 3.7 Current Signatures of Bystander T Cells Do Not Fully Discriminate the Tumor-Specific Reactive TIL Population *In Situ*

**Figure 4:**
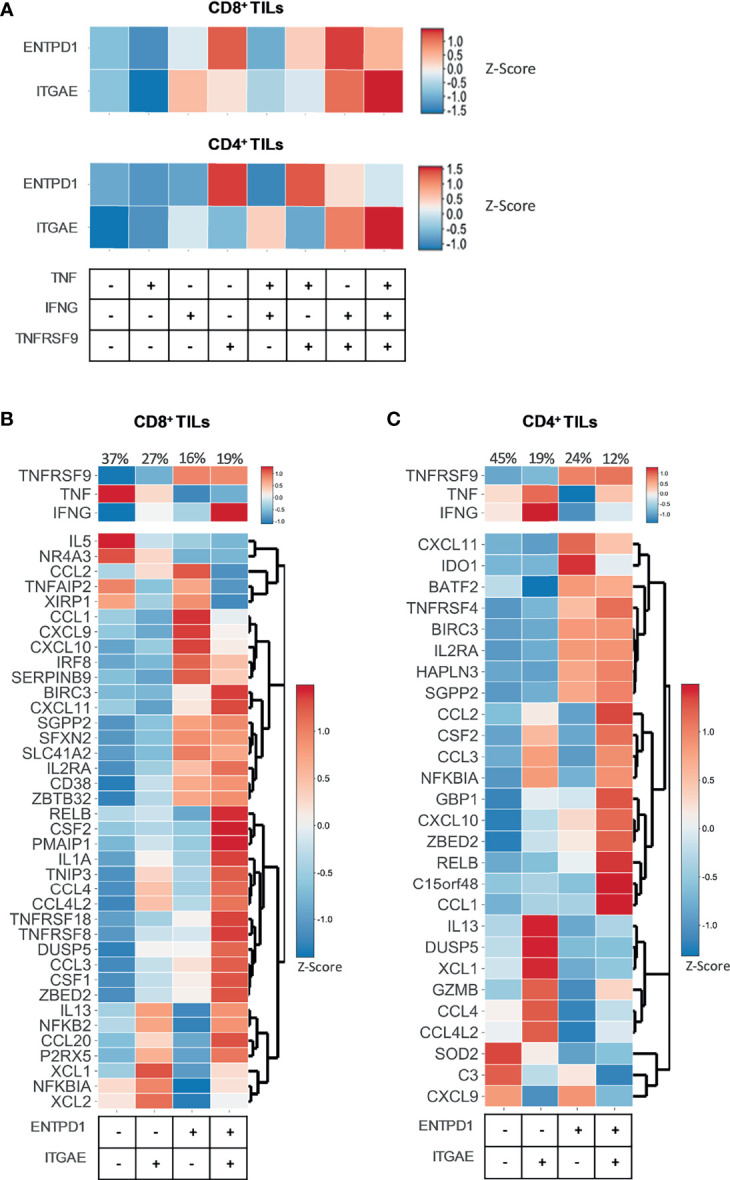
*ENTPD1* and *ITGAE* do not fully discriminate the tumor-specific reactive TIL population *in situ*. Heatmaps showing data from 12748 single CD8^+^ TILs and 10654 single CD4^+^ TILs isolated from tumor tissue of four different cancer types (non-small-cell lung cancer, hepatocellular carcinoma, colorectal cancer, and melanoma). Six selected scRNAseq datasets from public repositories were re-analyzed to produce this figure (see **Supplementary Table 2**). **(A)** The heatmaps show the expression (Z-score) of the genes *ENTPD1* and *ITGAE* within eight functional clusters of CD8^+^ and CD4^+^ TILs identified *in situ* through the differential expression of *TNFRSF9, TNF*, and *IFNG*. Every functional cluster represents a different combination of *TNFRSF9, TNF*, and *IFNG* expression. The expression of *ENTPD1* and *ITGAE* was heterogeneous across the different clusters, with *ENTPD1* expression being primarily associated with *TNFRSF9* expression. **(B, C)** The heatmaps show the expression (Z-score) of *TNFRSF9, TNF*, and *IFNG* and of the genes belonging to the **(B)** CD8 and **(C)** CD4 tumor-specific activation gene sets within four clusters of CD8^+^ and CD4^+^ TILs identified *in situ* through the differential expression of *ENTPD1* and *ITGAE*. Each cluster represents a different combination of *ENTPD1* and *ITGAE* expression. Despite most genes being highly expressed in *ENTPD1^+^* and/or *ITGAE^+^* positive clusters, the expression of some genes included in the activation gene sets was observed within the *ENTPD1^-^ ITGAE^-^* cluster. CD39 and CD103 are useful, but not sufficient to discriminate the entire repertoire of tumor-specific reactive TILs *in situ*.

CD39 and CD103 have largely been described as markers denoting tumor-specificity (1, 2, 46). Therefore, we sought to evaluate whether *ENTPD1* and *ITGAE*, respectively coding for CD39 and CD103, were expressed by the functional clusters identified *in situ* through the differential expression of *TNFRSF9, TNF*, and *IFNG*. The expression of both genes was heterogeneous across the different clusters, with *ENTPD1* expression being primarily associated with *TNFRSF9* expression (**Figure 4A**). To further investigate this issue, we studied the expression of *TNFRSF9, TNF*, *IFNG*, and our activation gene sets (**Supplementary Table 5**) across *ENTPD1^+/-^* and *ITGAE^+/-^* clusters (**Figures 4B, C**). Despite most genes being highly expressed in *ENTPD1^+^* and/or *ITGAE^+^* positive clusters, we could still observe the expression of genes such as *TNF* or *IL5*, for the CD8^+^ compartment, or *CXCL9* or *SOD2*, for the CD4^+^ compartment, within the *ENTPD1^-^ ITGAE^-^* cluster, indicating that CD39 and CD103 may be useful but not sufficient to discriminate the entire repertoire of tumor-specific reactive TILs *in situ* (**Figures 4B, C**).

## 4 Discussion

By developing and validating a rapid method detecting the majority of the real-time tumor-specific reactive CD8^+^ and CD4^+^ TIL repertoire *in vitro*, we have addressed a major ongoing issue in cancer immune-monitoring. Our protocol concurrently detecting intracellular CD137, TNF, and IFNγ by flow cytometry identified the majority of the presumed tumor-specific reactive TIL repertoire in the TME. This method combines widely available assays within a single novel protocol, thus allowing rapid adoption in most cancer immunology laboratories worldwide and potentially setting a new standard. However, due to its simplicity this combined assay does not consider the phenotype, exhaustion status, or specific antigen recognized by tumor-reactive TILs. Hence, while it can be advantageous when these parameters are irrelevant, it should be used with caution if a deep characterization of TIL functionality is desired.

Our optimized *in vitro* set-up revealed multiple distinct functional clusters of TILs expressing heterogeneous activation markers and functions on a protein level, a finding supported by recent data demonstrating that most tumor-specific TILs expanded from renal cell carcinoma express CD137, but may lack significant expression of common effector molecules (47). Similar heterogeneous functional clusters were found in the TME through re-analysis of publicly available T cell scRNAseq data from fresh tumor tissue; however, the mechanisms responsible for the multiple observed functional populations are unclear and were not fully addressed in this study. Several plausible explanations could be hypothesized, including a heterogeneous exhaustion/dysfunctional status of activated TILs (48, 49), varying TCR affinities (50), antigen concentration on the surface of autologous tumor cells (51), or the possibility that CD137^+^ TNF^-^ IFNγ^-^ TILs may be secreting other effector molecules. However, we cannot exclude that some of these functional clusters could represent bystander T cells, such as the CD137^-^ TNF^+^ IFNγ^-^ cluster, which was associated with a naïve profile. Notably, our data suggested that TILs expressing all three markers had a more pronounced exhausted/dysfunctional profile *in situ*, but were also associated with a high expression of genes coding for co-stimulatory and T cell effector molecules.

Previous studies suggested that a lower bystander to tumor-specific T cell ratio in the TME may correlate with better overall survival for cancer patients (2, 46, 52). It is therefore crucial that methods identifying tumor-specific reactive TILs are highly accurate. When we applied our activation gene sets to the CD8^+^ and CD4^+^ compartments *in situ*, we were not able to observe a clear pattern of association between the expression of our tumor-specific activation-related genes and the expression of common genes previously associated with tumor-specific TILs such as *ENTPD1* (CD39) and/or *ITGAE* (CD103), to the same degree that was observed with *TNFRSF9*, *TNF*, and/or *IFNG*. Hence, our results suggested a combination of *TNFRSF9*, *TNF*, and/or *IFNG* being superior to current methods to identify tumor-reactive TILs *in situ*. However, our data do not exclude that some of the functional clusters identified by *TNFRSF9*, *TNF*, and *IFNG*, could also represent bystander TILs. Notably, the literature has so far defined bystander T cells as CD39^-^ (1), but this definition does not exclude that a fraction of the tumor-specific T cells could also be CD39^-^. Kortekaas et al., 2020 *(*53) observed that a weak response against tumor antigens could be identified in CD39^-^ sorted CD4^+^ TILs, and tumor-reactive TILs can be found within a CD39^-^ CD69^-^ population in TILs expanded for adoptive T cell therapy (54).

Our study presents some important limitations. All *in situ* analyses have been carried out on RNA data, the correlation of which to protein level data can be challenging to establish. Additionally, we used expanded TILs for the co-culture assays and to generate the tumor-specific activation gene sets. The functional properties of TILs can be affected by *in vitro* expansion and could therefore differ from those of TILs *in vivo* or in scRNAseq data from fresh tumor tissue. We also cannot exclude that additional activation markers could be upregulated *in vivo* in tumor-specific reactive TILs; alternatively, TILs may acquire the ability to upregulate activation markers *via* culturing. Lastly, all co-culture assays presented in this study were performed after IFNγ exposure of the TCLs, leading to an MHC Class I and II upregulation that might not represent the same level of IFNγ-induced MHC upregulation happening *in vivo*.

In conclusion, we provide a straightforward method based on the simultaneous *in vitro* detection of CD137, TNF, and IFNγ by flow cytometry. This method improved the identification of tumor-specific reactive TILs *in vitro*. Of note, the simultaneous *in situ* detection of the corresponding genes, *TNFRSF9, TNF*, and *IFNG*, could represent a rapid and effective strategy for the identification and future functional characterization of the majority of the tumor-specific reactive TIL repertoire in scRNAseq data. Its potential applications are intriguing, and further exploration of this method is highly warranted.

## Data Availability Statement

The original contributions presented in the study are included in the article/**Supplementary Material**. Further inquiries can be directed to the corresponding author. One of the datasets presented in this article is not readily available due to personal data and patient confidentiality protection obligations. Requests to access the datasets should be directed to the corresponding author, and access to the data will be provided according to institutional policies and applicable laws.

## Author Contributions

Conceptualization: AD, CAC, MD; Supervision: MHA, IC, GJ, ZS, IMS, MD; Formal analysis: AD, CAC, SK, KP, ML, SS, HDR, MP, AG, AK, MN, MCWW; Data curation: AD, CAC, SK, KP, ML, SS, KH, MD; Statistical analysis: AD, CAC, SS, KP, ML; Data visualization: AD, CAC, SK, KP; Writing-original draft: AD, CAC, SK, MD; Resources supply and funding acquisition: MHA, IC, GJ, ZS, IMS, MD; All authors critically revised and edited the manuscript and approved it for final submission.

## Funding

This work was supported by the by The Danish Cancer Society under Grants R180-A11339, R184-A11806, and R204-A12535; the Lundbeck Foundation under Grants R233-2016-3728, R286-2018-991, and R307-2018-3636; the Independent Research Fund Denmark under Grant 8045-00067B; the Danish National Board of Health under Grant 4-1612-236/8 “Empowering Cancer Immunotherapy in Denmark”; the National Research, Development and Innovation Fund of Hungary under Grant Project no. FIEK_16-1-2016-0005; the Herlev and Gentofte Hospital Research Council under Clinician-Scientist Grant.

## Conflict of Interest

MD has received honoraria for lectures from Roche and Novartis (past two years). IMS has received honoraria for consultancies and lectures from Novartis, Roche, Merck, and Bristol-Myers Squibb; a restricted research grant from Novartis; and financial support for attending symposia from Bristol-Myers Squibb, Merck, Novartis, Pfizer, and Roche. HDR is currently an employee at Novo Nordisk.

The remaining authors declare that the research was conducted in the absence of any commercial or financial relationships that could be construed as a potential conflict of interest.

## Publisher’s Note

All claims expressed in this article are solely those of the authors and do not necessarily represent those of their affiliated organizations, or those of the publisher, the editors and the reviewers. Any product that may be evaluated in this article, or claim that may be made by its manufacturer, is not guaranteed or endorsed by the publisher.
