# Supplementary material for: Rapid Identification of the Tumor-Specific Reactive TIL Repertoire via Combined Detection of CD137, TNF, and IFNγ, Following Recognition of Autologous Tumor-Antigens
Source: Front Immunol. 2021 Oct 11;12:705422. doi: 10.3389/fimmu.2021.705422 (PMC8543011; doi:10.3389/fimmu.2021.705422)
Supplement: Supplementary file 1 [file DataSheet_1.docx]

Supplementary Material

# Supplementary Methods

## Antibodies

The following antibodies were used: anti-HLA–DR, DP, DQ (FITC, Clone Tu39, Cat No 555558, BD Biosciences), anti-CD4 (FITC, Clone SK3, Cat No 345768, BD Biosciences), anti-CD137 (PE, Clone 4B4-1, Cat No 555956, BD Biosciences), anti-CD3 (PE-CF594, Clone UCHT1, Cat No 562280, BD Biosciences), anti-CD8 (PerCP, Clone SK1, Cat No 345774, BD Biosciences), anti-IFNγ (PE-Cy7, Clone B27, Cat No 557643, BD Biosciences), anti-HLA-ABC (APC, Clone G46-2.6, Cat No 555555, BD Biosciences), anti-CD137 (APC, Clone 4B4-1, Cat No 309810, BioLegend, San Diego, CA, USA), anti-TNF (APC, Clone MAb11, Cat No 554514, BD Biosciences), Live/Dead Fixable Dead Cell Stain Near-IR NIR (APC-Cy7, Cat No L34976, Thermo Fisher Scientific), anti-CD107a (BV421, Clone H4A3, Cat No 562623, BD Biosciences), anti-CD56 (BV510, Clone NCAM16.2, Cat No 563041, BD Biosciences), anti-CD8 (Qdot605, Clone 3B5, Cat No Q10009, Thermo Fisher Scientific), anti-CD4 (BV711, Clone SK3, Cat No 563028, BD Biosciences).

The panels shown in Supplementary Table 1 were used to evaluate the differential expression of CD137 on activated T cells in the presence or absence of BFA and/or MN, and to evaluate intracellular CD137 staining in combination with the detection of TNF, IFNγ, and CD107a. For sorting of antigen-specific and enriched tumor-reactive TILs, in house produced peptide–MHC multimers (PE or APC or BV421 conjugated), anti-CD4 FITC, anti-CD8 PerCP, and anti-CD137 APC antibodies were used.

## Reanalysis of T cell transcriptomics single-cell data from public repositories

Patient Mel78 from Jerby-Arnon *et al*. 2018(1) was excluded from the analyses due to the extremely low number of T cells isolated, whereas no CD4^+^ T cells could be identified from patient P1207 from Zhang *et al*. 2018(2) after data processing. Raw count data and metadata matrices for the selected datasets were downloaded from the Gene Expression Omnibus (GEO)(3) or requested directly from the authors in case of unavailability on GEO (Supplementary Table 2).

For the six selected datasets, conflicting gene IDs were provided by the corresponding research groups. The HUGO Gene Nomenclature Committee (<https://www.genenames.org/download/custom/>, downloaded on 07/06/2020) was used to convert given gene IDs to Entrez Gene IDs for all datasets to unify the data. Of the six datasets, three(2, 4, 5) provided only “*CCL4L1”* as the gene ID for all isoforms deriving from *CCL4L1* and *CCL4L2.* These were changed to “*CCL4L2”* for consistency with our in-house bulk RNA sequencing data, which contained only *CCL4L2* and not *CCL4L1*, during the downstream analysis. In the two datasets from Jerby-Arnon *et al*. 2018(1), the same average expression was provided for both *CCL4L1* and *CCL4L2*, suggesting that both gene IDs represented the data pertaining to all isoforms deriving from *CCL4L1* and *CCL4L2.* Lastly, for the dataset from Sade-Feldman *et al*. 2019(6), distinct data were provided under the gene IDs “*CCL4L1”* and “*CCL4L2”* (Supplementary Table 3), and therefore the sum of the expression values of the two genes, represented as “*CCL4L2*” was used in our analyses. The R (version 3.6.2) function merge was then used to create a single data and metadata matrix from all individual data and metadata matrices. We kept only cells isolated from tumor tissues for further downstream analysis and removed those expressing <500 genes. Genes expressed in less than three cells were also removed. The final merged data matrix contained a total of 27583 cells and 21378 genes. Normalization of the data was performed using the SCTransform function from the Seurat R package(7, 8) with batch_var=Patient to regress out latent variables.

For the identification of CD4^+^ and CD8^+^ T cells, the following criteria were consecutively applied:

(i) Based on *CD3D*, *CD3E*, *CD3G* expression:

*T cells = CD3D+ OR CD3E+ OR CD3G+*

(ii) Based on *CD4*, *CD8A* and *CD8B* expression:

*CD4^+^ T cells = CD4+ AND CD8A- AND CD8B-*

*CD8^+^ T cells = CD4- AND (CD8A+ OR CD8B+)*

Post-filtering, 12748 single CD8^+^ TILs and 10654 single CD4^+^ TILs were identified, and a total of 5181 “unknown” cells were excluded from further analyses.

Subsequently, CD8^+^ and CD4^+^ TILs were classified as *TNFRSF9*^+/-^, *TNF*^+/-^, *IFNG*^+/-^, *ENTPD1*^+/-^, and *ITGAE*^+/-^. Cells were considered “positive” or “negative” for expression of a specific gene according to manually set thresholds based on bimodal distribution of individual gene expression. The merged normalized data were additionally integrated using the IntegrateData function from Seurat for data visualization using tSNE plots. All heatmaps were based on cluster-averaged gene expression to account for cell-to-cell transcriptomic stochastics. Data autoscaling was used for the visualization.

# Supplementary Tables

| **Supplementary Table 1: Flow-cytometry antibody panels.** | | | | |
| --- | --- | --- | --- | --- |
| **Panel purpose** | **Staining phase** | **Target** | **Fluorochrome** | **Purpose** |
| **Evaluation of CD137 expression in the presence of BFA and/or MN** | Surface staining | CD3 | PE-CF594 | T cell Lineage |
|  |  | CD56 | BV510 | NK cell lineage |
|  |  | CD4 | BV711 | T cell Lineage |
|  |  | CD8 | Qdot605 | T cell Lineage |
|  |  | CD137 | APC | Activation |
|  |  | Dead cells | APC-Cy7 | Live/dead |
|  | Intracellular staining | CD137 | PE | Activation |
| **Evaluation of the combined detection of CD137, TNF, IFNγ, and CD107a** | Co-culture | CD107a | BV421 | Degranulation |
|  | Surface staining | CD4 | BV711 | T cell Lineage |
|  |  | CD8 | Qdot605 | T cell Lineage |
|  |  | Dead cells | APC-Cy7 | Live/dead |
|  | Intracellular staining | TNF | APC | Activation |
|  |  | IFNγ | PE-Cy7 | Activation |
|  |  | CD137 | PE | Activation |

| **Supplementary Table 2: Single-cell RNA sequencing datasets accession numbers (*in situ* data)** | | | |
| --- | --- | --- | --- |
| **Dataset** | **Title** | **Data source** | **Tumor Histology** |
| Guo *et al.* (4) | T cell landscape of non-small cell lung cancer revealed by deep single-cell RNA sequencing | GSE99254 | NSCLC |
| Zheng *et al.* (5) | Landscape of infiltrating T cells in liver cancer revealed by single-cell sequencing | GSE98638 | HCC |
| Zhang *et al.* (2) | Lineage tracking reveals dynamic relationships of T cells in colorectal cancer | GSE108989 | CRC |
| Jerby-Arnon *et al*.* (1) | A Cancer Cell Program Promotes T Cell Exclusion and Resistance to Checkpoint Blockade | GSE115978 | MM |
| Sade-Feldman *et al*. (6) | Defining T cell states associated with response to checkpoint immunotherapy in melanoma | Obtained directly from authors | MM |
| * Two datasets were obtained from this study. One of the datasets was previously published in Tirosh *et al*. (9) | |  |  |

| **Supplementary Table 3: Average gene expression of selected genes** | | | | | |
| --- | --- | --- | --- | --- | --- |
| **Dataset** | ***CCL4L1*** | ***CCL4L2*** | ***CD4*** | ***CD8A*** | ***CD8B*** |
| Guo *et al*. | 4,10 | - | 5,71 | 6,36 | 4,86 |
| Zhang *et al*. | 4,81 | - | 5,47 | 6,49 | 4,72 |
| Zheng *et al*. | 4,37 | - | 6,26 | 6,77 | 5,26 |
| Jerby1 (Tirosh *et al*.) | 3,82 | 3,82 | 3,94 | 5,63 | 4,05 |
| Jerby2 (Jerby-Arnon *et al*.) | 4,37 | 4,37 | 5,46 | 6,73 | 5,62 |
| Sade-Feldman *et al*. | 2,01 | 2,10 | 5,11 | 6,66 | 4,86 |

| **Supplementary Table 4: Mean Log_2_ Fold Change of common tumor-reactivity and specificity markers following tumor-specific activation (in-house bulk RNAseq data).** | | | | |
| --- | --- | --- | --- | --- |
| **Gene** | **CD8^+^ TILs** | | **CD4^+^ TILs** | |
|  | **Mean LFC** | **p-value** | **Mean LFC** | **p-value** |
| ***CD40LG*** | 0,07 | 0,80965 | -0,05 | 0,69156 |
| ***GZMB*** | **1,94** | **0,00003** | **1,82** | **0,00037** |
| ***IFNG*** | **3,97** | **0,00004** | **2,27** | **0,00166** |
| ***IL2*** | 0,44 | 0,08292 | 0,38 | 0,09497 |
| ***LAMP1*** | -0,01 | 0,88712 | 0,05 | 0,56125 |
| ***TNF*** | **1,02** | **0,00046** | **1,50** | **0,00101** |
| ***TNFRSF9*** | **5,35** | **< 0,00001** | **2,92** | **0,00008** |
| Significant values marked in bold | |  |  |  |

| **Supplementary Table 5: Tumor-specific activation gene sets (in-house bulk RNAseq data)** | |
| --- | --- |
| **CD8** | **CD4** |
| LFC ≥ 2 and p-value <0.01 | LFC ≥ 1.4 and p-value <0.01 |
| *BIRC3** | *BATF2* |
| *CCL1** | *BIRC3** |
| *CCL2** | *C15orf48* |
| *CCL20* | *C3* |
| *CCL3** | *CCL1** |
| *CCL4** | *CCL2** |
| *CCL4L2** | *CCL3** |
| *CD38* | *CCL4** |
| *CRTAM* | *CCL4L2** |
| *CSF1* | *CSF2** |
| *CSF2** | *CXCL10** |
| *CXCL10** | *CXCL11** |
| *CXCL11** | *CXCL9** |
| *CXCL9** | *DUSP5** |
| *DUSP5** | *GBP1* |
| *IL13** | *GZMB* |
| *IL1A* | *HAPLN3* |
| *IL2RA** | *IDO1* |
| *IL5* | *IL13** |
| *IRF8* | *IL2RA** |
| *LTA* | *NFKBIA** |
| *NFKB2* | *RELB** |
| *NFKBIA** | *SGPP2* |
| *NR4A3* | *SOD2* |
| *P2RX5* | *TNFRSF4* |
| *PMAIP1* | *XCL1** |
| *RELB** | *ZBED2** |
| *SERPINB9* |  |
| *SFXN2* |  |
| *SGPP2* |  |
| *SLC41A2* |  |
| *TNFAIP2* |  |
| *TNFRSF18* |  |
| *TNFRSF8* |  |
| *TNIP3* |  |
| *XCL1** |  |
| *XCL2* |  |
| *XIRP1* |  |
| *ZBED2** |  |
| *ZBTB32* |  |

*genes shared by the CD8 and CD4 gene sets

# Supplementary Figures


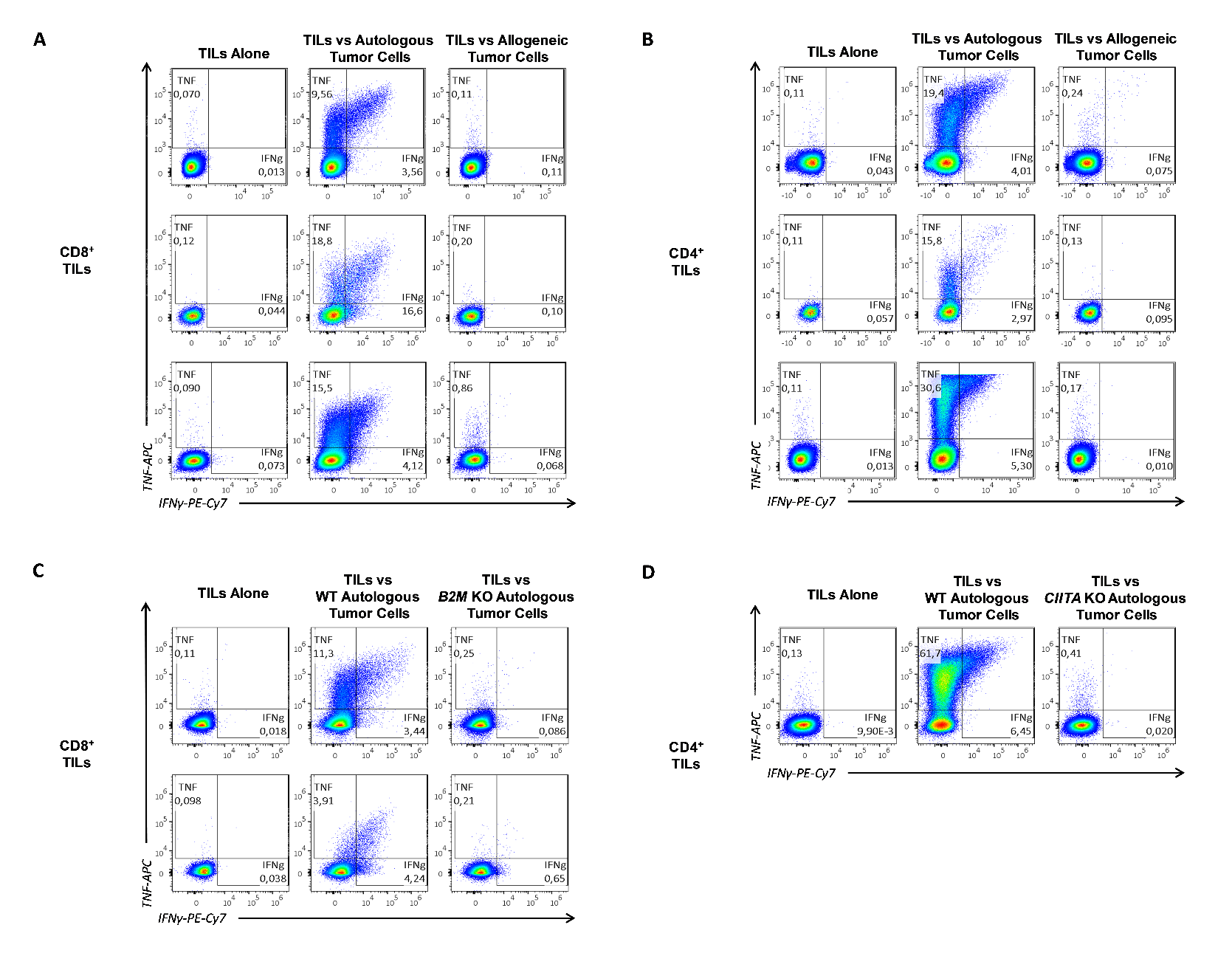


**Supplementary Figure 1. Matched pairs of TILs and TCLs were selected based on lack of TIL reactivity after co-culture with control TCLs *in vitro*. (A-B)** To study gene and functional marker upregulation in TILs after tumor-specific activation, a pre-screening was performed using TILs and multiple allogeneic TCLs to rule out any unspecific T cell activation by irrelevant TCLs. Allogeneic TCLs showing a lack of reactivity based on TNF, IFNγ, and CD107a expression were used as a negative control. Only TILs where no upregulation of functional markers was detected when co-culturing with at least one allogeneic TCL were used in this study. The dot plots show the intracellular expression of TNF and IFNγ in three representative (A) CD8^+^ TIL samples and three representative (B) CD4^+^ TIL samples following 8 hours of co-culture with none, autologous or allogeneic tumor cells. **(C-D)** To verify that the observed TIL activation was mediated by classical TCR-MHC recognition of autologous tumor-antigens, selected TIL samples were co-cultured with autologous TCLs whose MHC class I or class II expression was abrogated via *B2M* or *CIITA* KO, respectively. The dot plots show the intracellular expression of TNF and IFNγ in two representative (C) CD8^+^ TIL samples and one representative (D) CD4^+^ TIL sample following 8 hours of co-culture with none, autologous wild-type or autologous MHC-KO tumor cells.


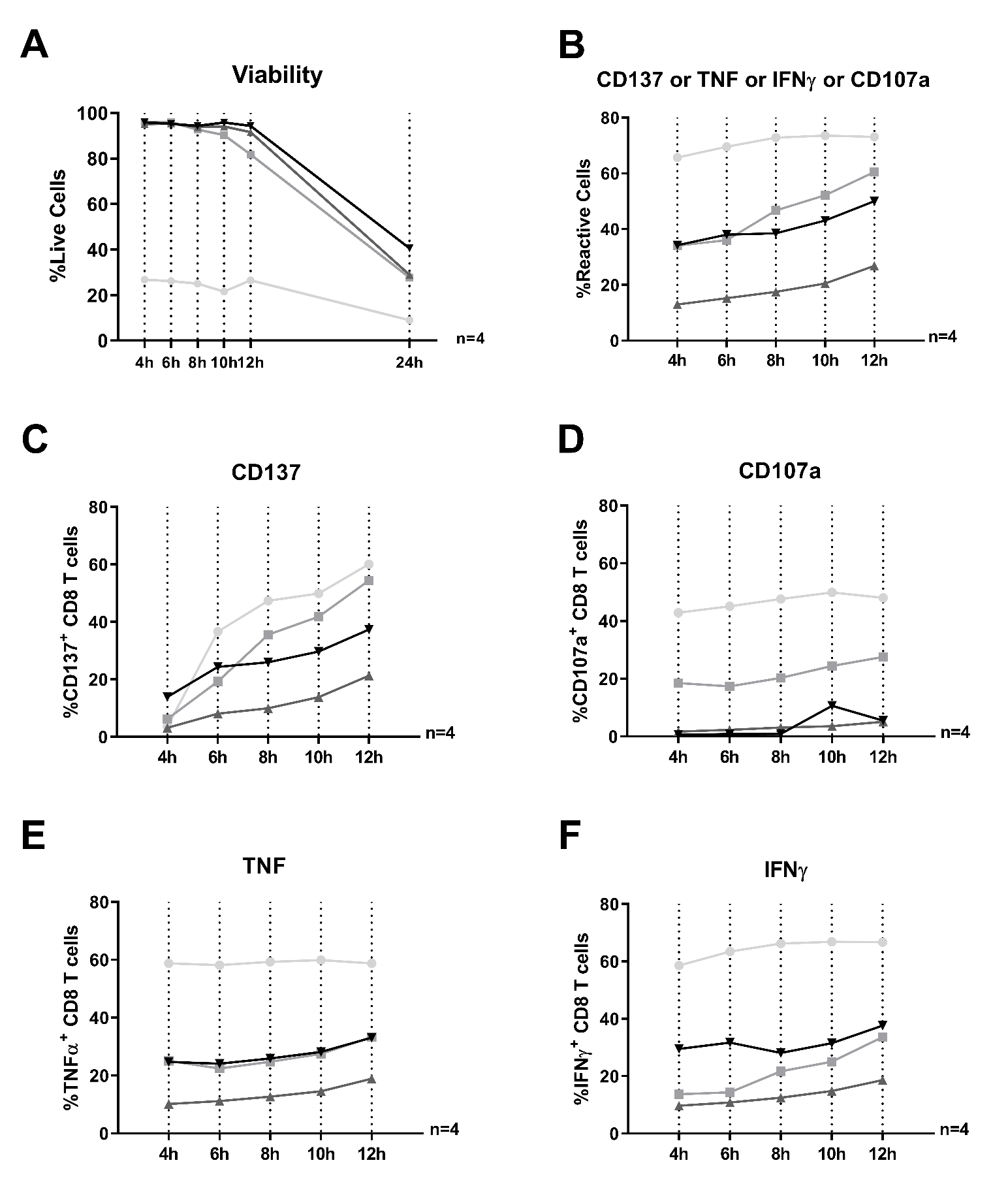


**Supplementary Figure 2. Expression kinetics of various activation/functional markers in bulk CD8^+^ TILs after co-culture with autologous tumor cells.** (**A**) The panel shows the TIL viability at different time points after co-culture with autologous tumor cells in the presence of both BFA and MN (n=4). (**B-F**) The panels show the expression of (B) CD137 or TNF or IFNγ or CD107a, (C) CD137 alone, (D) CD107a alone, (E) TNF alone, and (F) IFNγ alone in CD8^+^ TILs at different time points after co-culture with autologous tumor cells in the presence of both BFA and MN (n=4). Bulk TILs were used to produce the flow cytometry data presented in this figure.


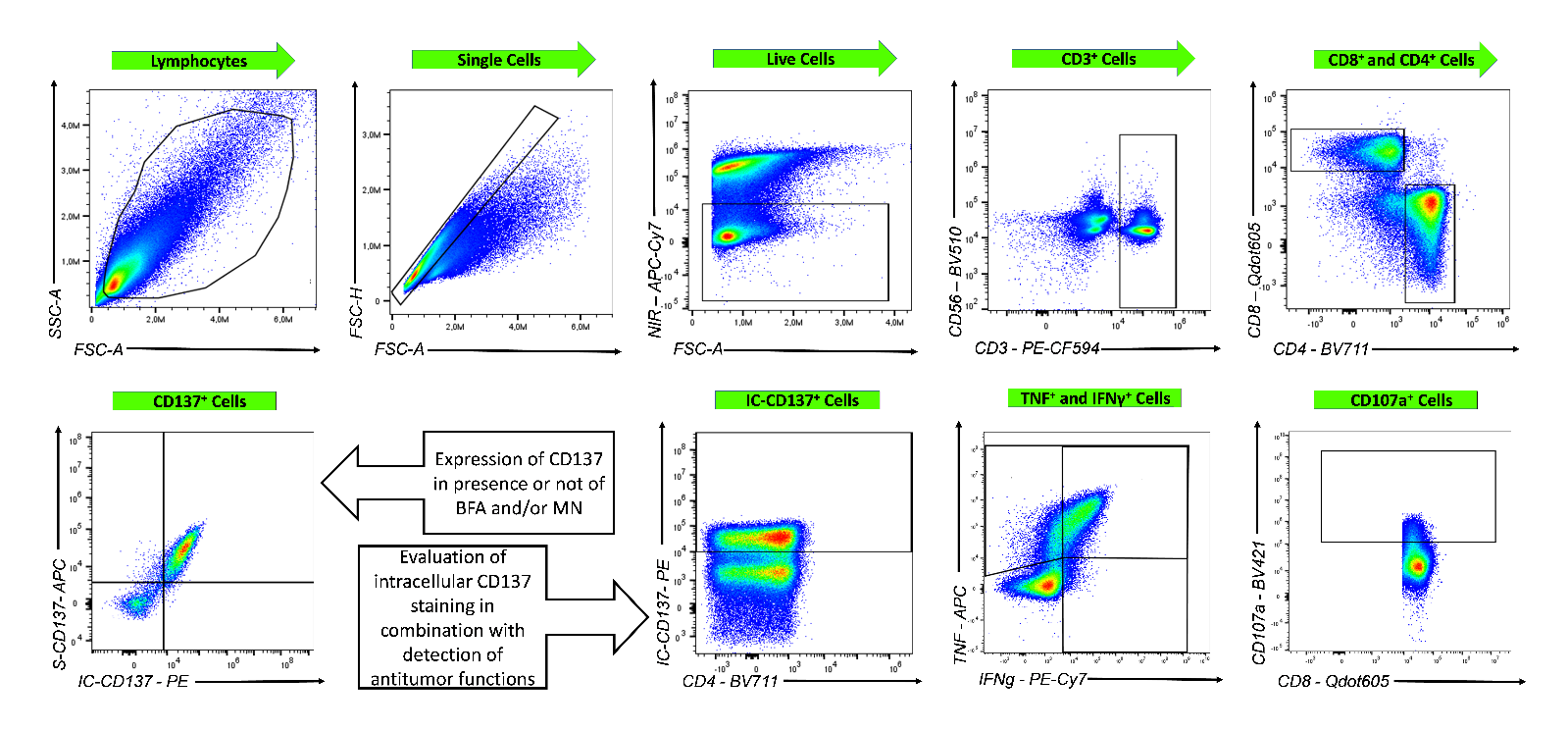


**Supplementary Figure 3. Gating strategy used to identify CD137^+^ and Antitumor Function^+^ T cells.** Lymphocytes were selected based on a plot of FSC-A vs. SSC-A and doublets were removed by gating FSC-A vs. FSC-H. Subsequently, cells negative for Live/Dead Fixable Dead Cell Stain Near-IR (NIR) were gated as live cells, and CD3^+^ T cells were gated in a plot of CD3 vs. CD56. CD4^+^ and CD8^+^ populations were identified in a CD4 vs CD8 plot. In tests evaluating the differential expression of CD137 on activated TILs in the presence or absence of BFA and/or MN, CD137^+^ TILs were gated in a plot of surface CD137 (S-CD137) vs. intracellular CD137 (IC-CD137). Boolean gating “S-CD137 OR IC-CD137” was performed to obtain the Total CD137^+^ population. In tests evaluating CD137 staining in combination with the detection of TNF, IFNγ, and CD107a, CD137^+^ TILs were gated in a plot of IC-CD137 vs. CD4 or CD8. TNF^+^ and IFNγ^+^ populations were gated in a plot of TNF vs. IFNγ. CD107a^+^ TILs were gated in a plot of CD107a vs. CD4 or CD8. Boolean gating was performed to obtain “Antitumor function^+^ TILs” and “Total Reactive TILs” subpopulations.


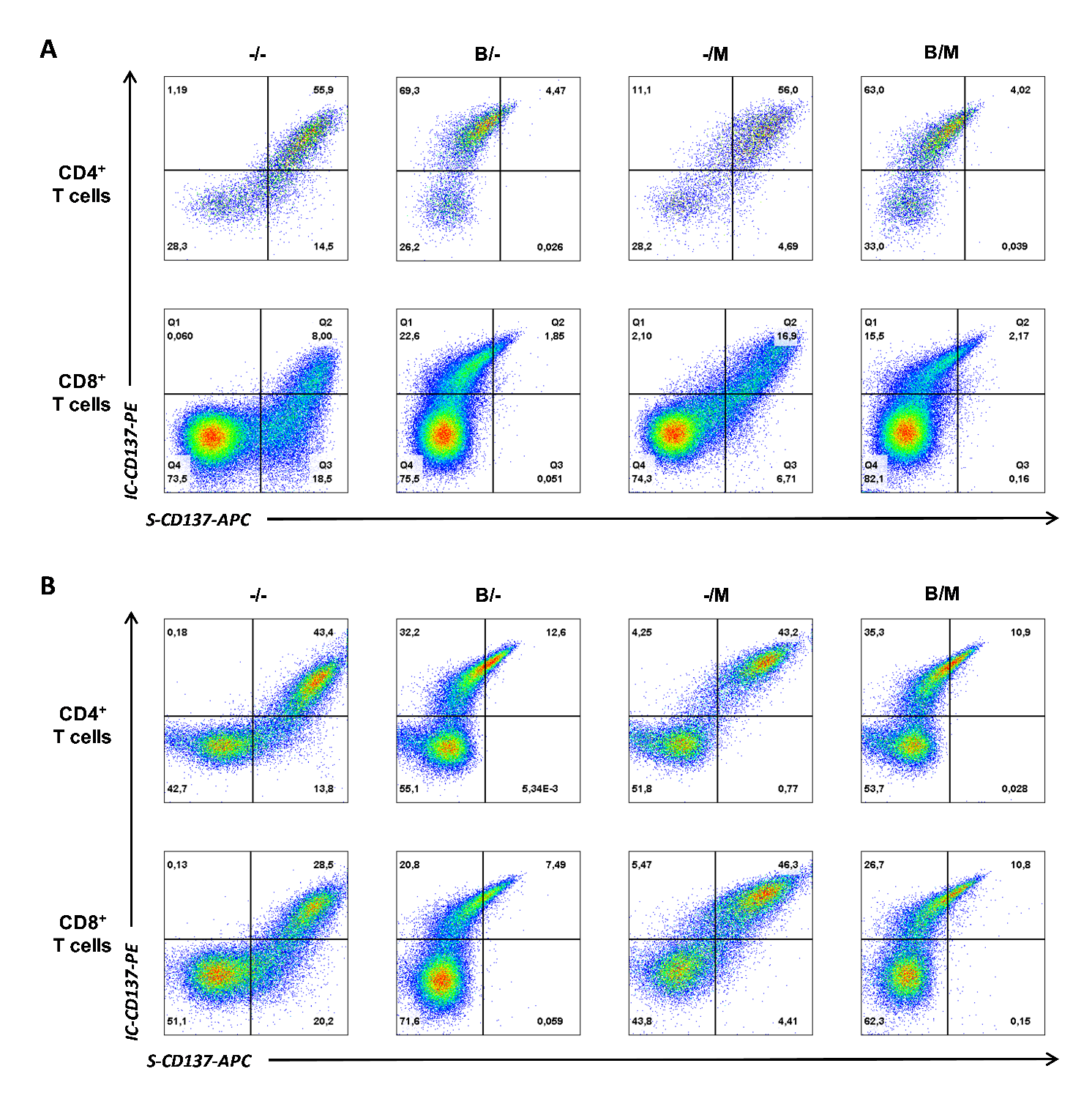


**Supplementary Figure 4. Surface and intracellular CD137 expression after exposure to protein transport inhibitors. (A-B)** Dot plots from two exemplary bulk TIL samples showing surface (S-CD137) and intracellular (IC-CD137) expression of CD137 in CD8^+^ and CD4^+^ TILs following 8 hours of co-culture with autologous tumor cells in the presence of different combinations of BFA and MN. B = brefeldin A, BFA;M = monensin, MN

**
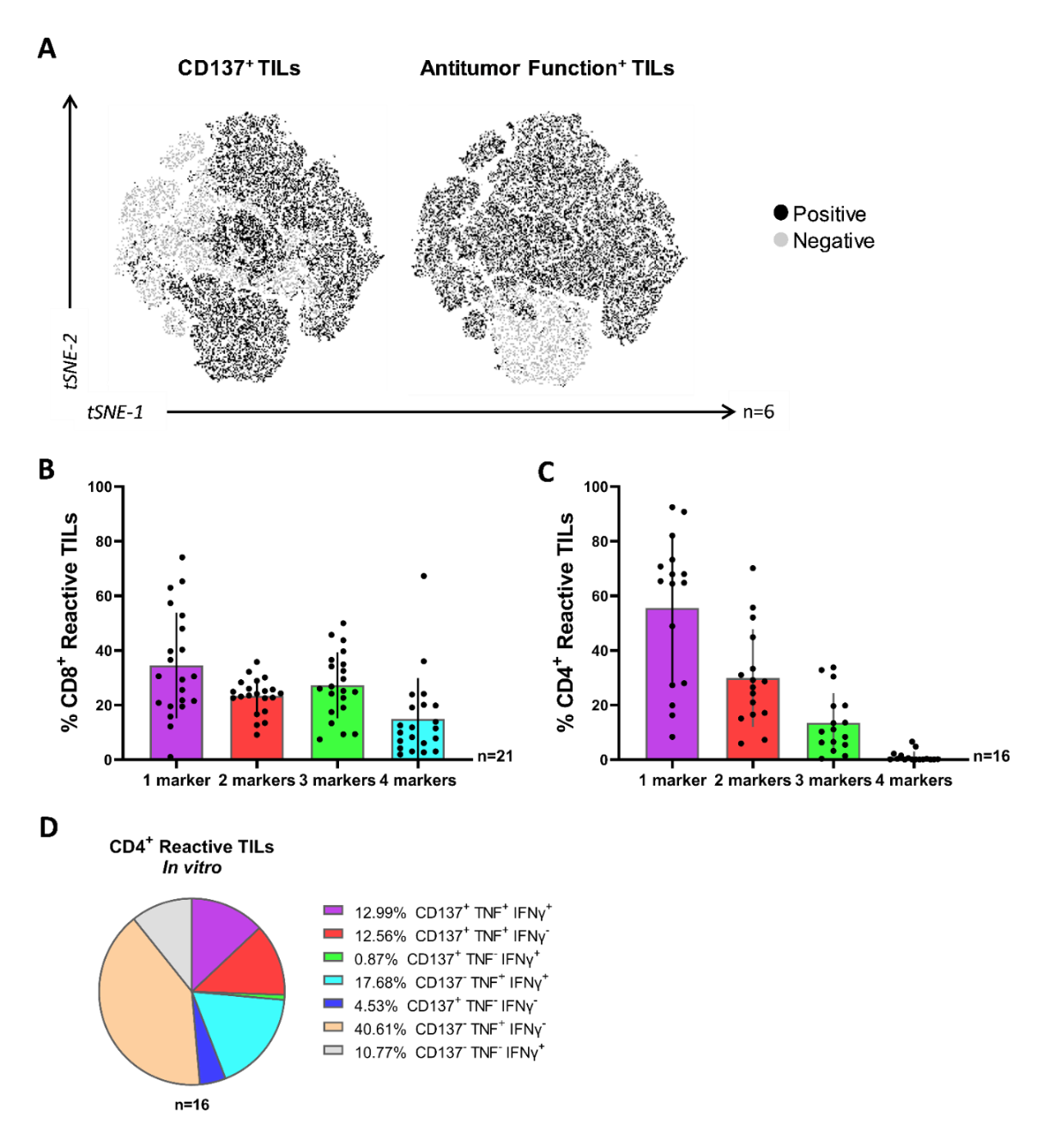
**

**Supplementary Figure 5. Optimized detection of tumor-specific reactive TILs *in vitro* identifies multiple functional clusters.** (**A**) t-SNE plots visualizing CD137^+^ TILs (left panel) or Antitumor Function^+^ TILs (right panel) within the global CD8^+^ tumor-specific reactive TIL repertoire, identified via combined detection of CD137, TNF, IFNγ, and CD107a by intracellular staining(n=6). Black indicates cells expressing the relevant marker/markers; light grey indicates other tumor-specific reactive cells. TNF, IFNγ, and CD107a were defined as “Antitumor functions”, therefore, TILs were defined Antitumor Function^+^ if staining positive for at least one of TNF, IFNγ, and CD107a. (**B-C**) Relative distribution of tumor-specific reactive (B) CD8^+^ and (C) CD4^+^ TILs generating 1, 2, 3, or 4 of the markers analyzed (CD137, TNF, IFNγ, and CD107a). Black bars represent standard deviations. Columns illustrate mean values. TILs were gated on cells expressing at least one of the four markers analyzed (CD137, TNF, IFNγ, and CD107a). (**D**) Relative distribution of the seven combinations of the three markers of interest (CD137, TNF, and IFNγ) within tumor-specific reactive CD4^+^ TILs. Every pie chart slice represents a different combination of CD137, TNF, and IFNγ. TILs were gated on cells expressing at least one of the three markers analyzed (CD137, TNF, and IFNγ). The pie chart illustrates mean values. Bulk TILs or enriched CD8^+^ and CD4^+^ bulk TILs were used to produce the intracellular staining data presented in this figure.

**Supplementary Figure 6. Tumor-specific activation gene set upregulation in antigen-specific or enriched tumor-reactive CD8^+^ TILs.** In-house bulk RNA sequencing data from two autologous tumor antigen-specific CD8^+^ TIL samples and three enriched tumor-reactive CD8^+^ TIL samples. The heatmap shows the LFCs in gene expression (CD8 tumor-specific activation gene set) between TILs co-cultured with autologous tumor cells and TILs co-cultured with allogeneic tumor cells. The majority of the genes included in the CD8 tumor-specific activation gene set were upregulated in both the antigen-specific CD8^+^ TIL samples and the enriched tumor-reactive CD8^+^ TIL samples.


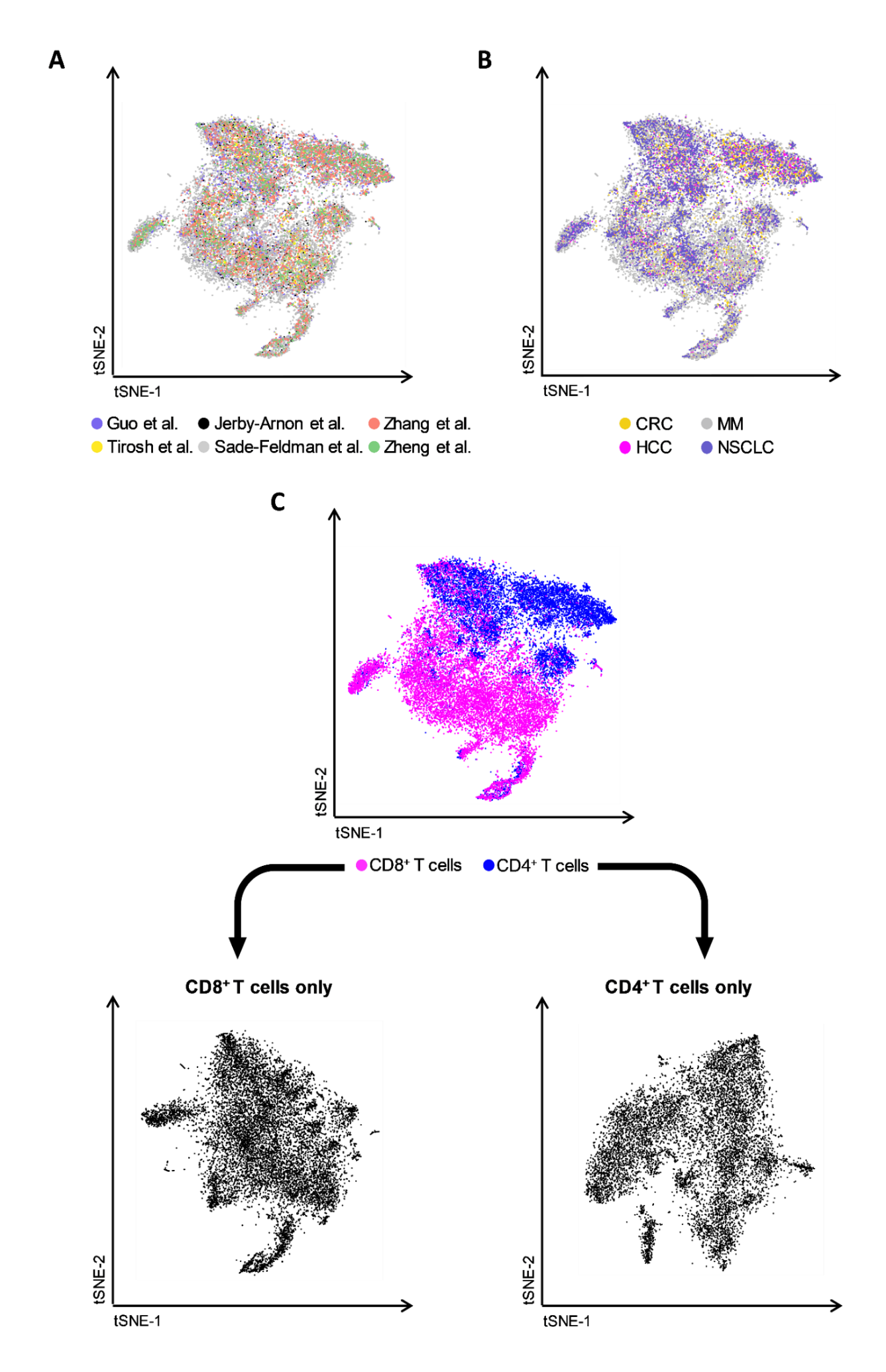


**Supplementary Figure 7. Processing of T cell transcriptomics single-cell data from public repositories. (A-C)** t-SNE plots showing 12748 single CD8^+^ T cells and 10654 single CD4^+^ T cells isolated from tumor tissue from **(A)** six individual datasets from five independent studies and from **(B)** four different tumor types: colorectal cancer (CRC), hepatocellular carcinoma (HCC), melanoma (MM) and non-small-cell lung cancer (NSCLC). **(C)** t-SNE plots visualizing CD8^+^ and CD4^+^ TILs within the total TIL population *in situ* or independently. Six selected scRNAseq datasets from public repositories were reanalyzed to produce this figure (see Supplementary Table 2).


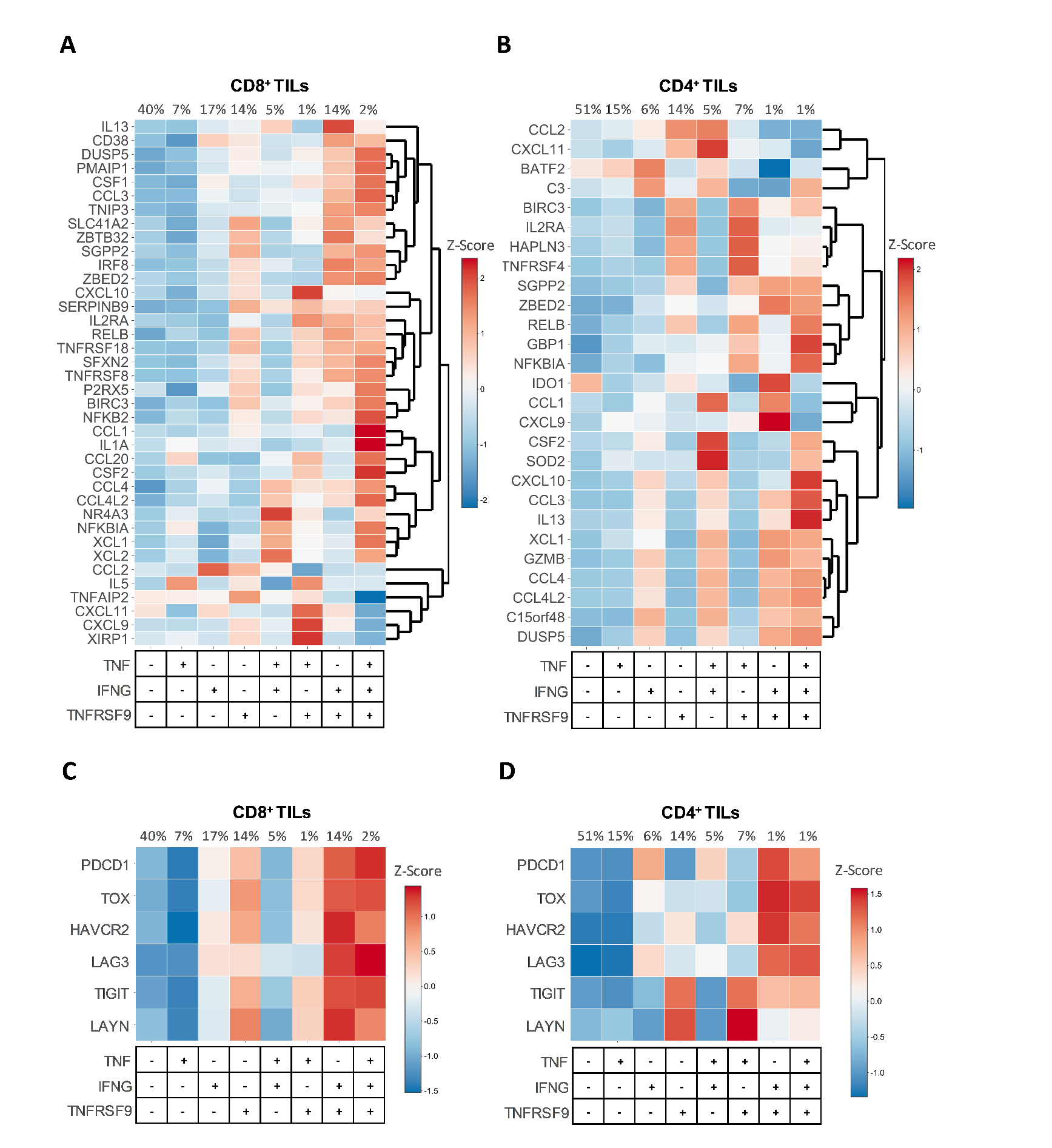


**Supplementary Figure 8. *TNFRSF9, TNF,* and *IFNG* expression identify multiple functional clusters of tumor-specific reactive TILs *in situ.*** Heatmaps showing data from 12748 single CD8^+^ TILs and 10654 single CD4^+^ TILs isolated from tumor tissue of four different cancer types (non-small-cell lung cancer, hepatocellular carcinoma, colorectal cancer, and melanoma). Six selected scRNAseq datasets from public repositories were reanalyzed to produce this figure (see Supplementary Table 2). (**A-B**) The heatmaps show the expression (Z-score) of the genes belonging to the **(A)** CD8 and **(B)** CD4 tumor-specific activation gene sets within eight functional clusters of CD8^+^ and CD4^+^ TILs identified *in situ* through the differential expression of *TNFRSF9, TNF,* and *IFNG*. Each functional cluster represents a different combination of *TNFRSF9, TNF,* and *IFNG* expression. Expression of the genes included in the activation gene sets increased as the positivity for the genes coding for the activation markers (CD137, TNF, or IFNγ) increased. (**C-D**) The heatmaps show the expression (Z-score) of genes commonly associated with T cell exhaustion/dysfunction within the eight functional clusters of **(C)** CD8^+^ and **(D)** CD4^+^ TILs identified *in situ* through the differential expression of *TNFRSF9, TNF,* and *IFNG*. The greatest level of exhaustion/dysfunction was observed in the clusters expressing *TNFRSF9* and *IFNG* or *TNFRSF9, TNF,* and *IFNG*. The gene with the highest level of association with exhaustion/dysfunction was *TNFRSF9*.


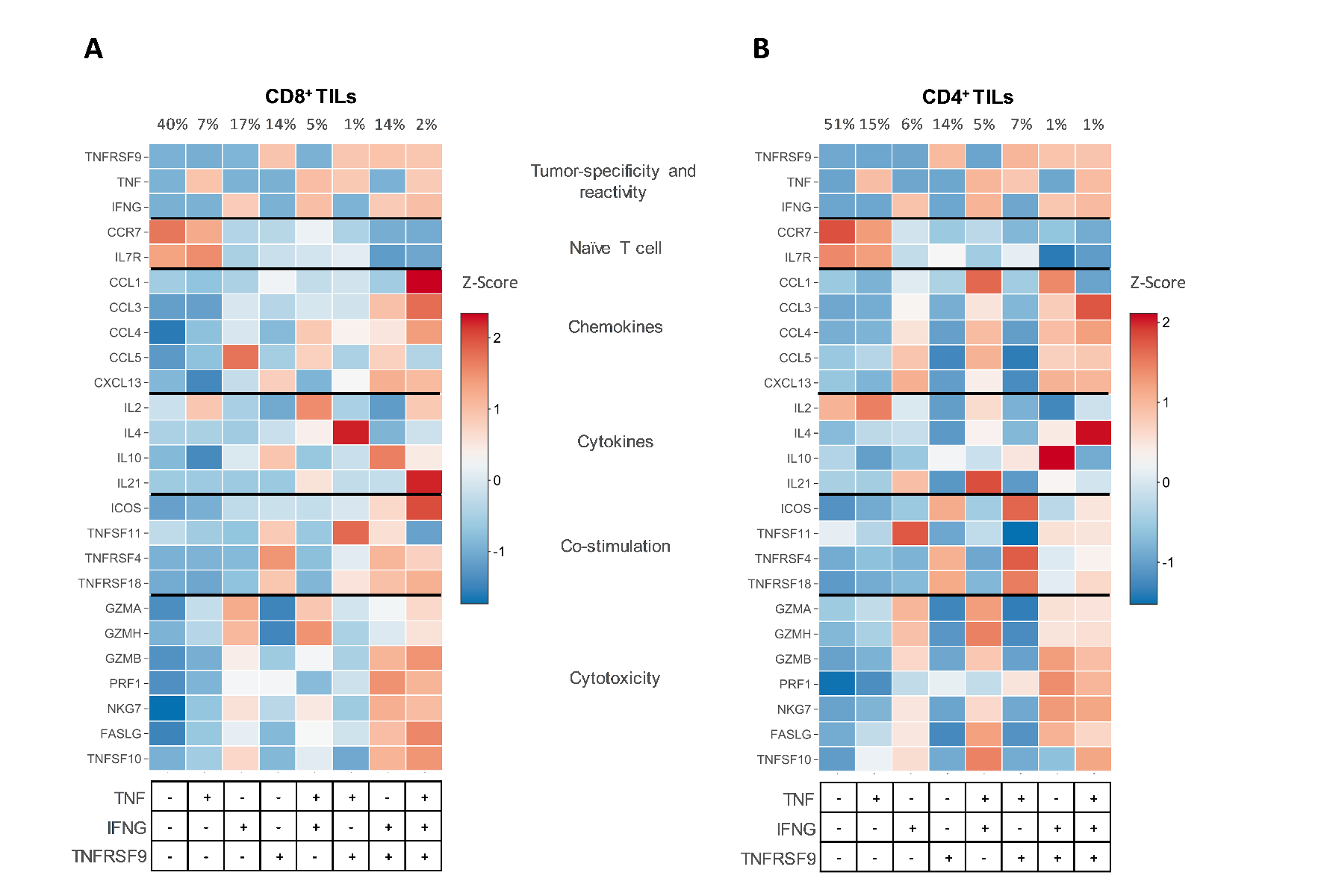


**Supplementary Figure 9. *TNFRSF9, TNF,* and *IFNG* expression is associated with the expression of genes coding for common co-stimulatory and T cell effector molecules *in situ.*** Heatmaps showing data from 12748 single CD8^+^ TILs and 10654 single CD4^+^ TILs isolated from tumor tissue of four different cancer types (non-small-cell lung cancer, hepatocellular carcinoma, colorectal cancer, and melanoma). Six selected scRNAseq datasets from public repositories were reanalyzed to produce this figure (see Supplementary Table 2). (**A-B**) The heatmaps show the expression (Z-score) of genes commonly associated with naïve T cells, and common T cell co-stimulatory or effector molecules within the eight functional clusters of **(A)** CD8^+^ and **(B)** CD4^+^ TILs identified *in situ* via the differential expression of *TNFRSF9, TNF,* and *IFNG*. The expression of genes coding for naïve T cell markers was observed in the Triple Negative cluster and in the cluster expressing *TNF* only. Expression of the genes associated with T cell co-stimulation and effector molecules increased as the positivity for the genes coding for the activation markers (CD137, TNF, or IFNγ) increased.

**References**

1. Jerby-Arnon L, Shah P, Cuoco MS, Rodman C, Su MJ, Melms JC, et al. A Cancer Cell Program Promotes T Cell Exclusion and Resistance to Checkpoint Blockade. Cell. 2018;175(4):984-97 e24.

2. Zhang L, Yu X, Zheng L, Zhang Y, Li Y, Fang Q, et al. Lineage tracking reveals dynamic relationships of T cells in colorectal cancer. Nature. 2018;564(7735):268-72.

3. Barrett T, Troup DB, Wilhite SE, Ledoux P, Rudnev D, Evangelista C, et al. NCBI GEO: mining tens of millions of expression profiles--database and tools update. Nucleic Acids Res. 2007;35(Database issue):D760-5.

4. Guo X, Zhang Y, Zheng L, Zheng C, Song J, Zhang Q, et al. Global characterization of T cells in non-small-cell lung cancer by single-cell sequencing. Nat Med. 2018;24(7):978-85.

5. Zheng C, Zheng L, Yoo JK, Guo H, Zhang Y, Guo X, et al. Landscape of Infiltrating T Cells in Liver Cancer Revealed by Single-Cell Sequencing. Cell. 2017;169(7):1342-56 e16.

6. Sade-Feldman M, Yizhak K, Bjorgaard SL, Ray JP, de Boer CG, Jenkins RW, et al. Defining T Cell States Associated with Response to Checkpoint Immunotherapy in Melanoma. Cell. 2019;176(1-2):404.

7. Hafemeister C, Satija R. Normalization and variance stabilization of single-cell RNA-seq data using regularized negative binomial regression. Genome Biol. 2019;20(1):296.

8. Butler A, Hoffman P, Smibert P, Papalexi E, Satija R. Integrating single-cell transcriptomic data across different conditions, technologies, and species. Nat Biotechnol. 2018;36(5):411-20.

9. Tirosh I, Izar B, Prakadan SM, Wadsworth MH, 2nd, Treacy D, Trombetta JJ, et al. Dissecting the multicellular ecosystem of metastatic melanoma by single-cell RNA-seq. Science. 2016;352(6282):189-96.
